# Inhibition of SARS-CoV-2 coronavirus proliferation by designer antisense-circRNAs

**DOI:** 10.1093/nar/gkab1096

**Published:** 2021-11-24

**Authors:** Christina Pfafenrot, Tim Schneider, Christin Müller, Lee-Hsueh Hung, Silke Schreiner, John Ziebuhr, Albrecht Bindereif

**Affiliations:** Institute of Biochemistry, Justus Liebig University of Giessen, 35392 Giessen, Germany; Institute of Biochemistry, Justus Liebig University of Giessen, 35392 Giessen, Germany; Institute of Medical Virology, Justus Liebig University of Giessen, 35392 Giessen, Germany; Institute of Biochemistry, Justus Liebig University of Giessen, 35392 Giessen, Germany; Institute of Biochemistry, Justus Liebig University of Giessen, 35392 Giessen, Germany; Institute of Medical Virology, Justus Liebig University of Giessen, 35392 Giessen, Germany; Institute of Biochemistry, Justus Liebig University of Giessen, 35392 Giessen, Germany

## Abstract

Circular RNAs (circRNAs) are noncoding RNAs that exist in all eukaryotes investigated and are derived from back-splicing of certain pre-mRNA exons. Here, we report the application of artificial circRNAs designed to act as antisense-RNAs. We systematically tested a series of antisense-circRNAs targeted to the SARS-CoV-2 genome RNA, in particular its structurally conserved 5′-untranslated region. Functional assays with both reporter transfections as well as with SARS-CoV-2 infections revealed that specific segments of the SARS-CoV-2 5′-untranslated region can be efficiently accessed by specific antisense-circRNAs, resulting in up to 90% reduction of virus proliferation in cell culture, and with a durability of at least 48 h. Presenting the antisense sequence within a circRNA clearly proved more efficient than in the corresponding linear configuration and is superior to modified antisense oligonucleotides. The activity of the antisense-circRNA is surprisingly robust towards point mutations in the target sequence. This strategy opens up novel applications for designer circRNAs and promising therapeutic strategies in molecular medicine.

## INTRODUCTION

Coronaviruses are positive-strand RNA viruses with large polycistronic genomes of around 30 kb, which have been extensively studied since the 2003 SARS outbreak [severe acute respiratory syndrome, reviewed in (1)]. Following receptor-mediated coronavirus entry into susceptible host cells, the two large open reading frames (ORFs), 1a and 1b, located in the 5′-terminal two-thirds of the capped and polyadenylated coronavirus genome, are translated, resulting in two polyproteins that are processed by viral proteases to produce nonstructural proteins that direct viral RNA synthesis (2). Translation requires the 5′-untranslated region (UTR) of the genome RNA, which for SARS-CoV-2 comprises nucleotides 1–265 [for secondary structure models, see (3,4)].

Subsequently, the viral genome RNA serves as a template for negative-strand RNA synthesis. Two types of minus-strand RNAs are produced: first, full-length copies of the plus-strand RNA that are used as templates for the production of new genome RNAs, and second, a set of 5′-coterminal subgenomic (sg) minus-strand RNAs of varying length. The vast majority of sg-minus-strand RNAs carry at their 3′-end an ‘antileader’ sequence, i.e. a complement of the leader sequence located at the 5′-end of the genome, which they acquire in a process called ‘discontinuous extension’ of minus strands. The sg-minus strands serve as templates for the production of a nested set of sg-mRNAs that share a common 5′-leader sequence (75 nts in SARS-CoV-2, plus a few nucleotides upstream of the translation start codon of the first ORF in the respective mRNA). This unusual process of discontinuous (minus-strand) RNA synthesis is guided by pairing between complements of the conserved transcription-regulatory sequences (TRS) located upstream of the various ORFs in the 3′-region of the genome (‘body-TRS’) and the TRS located immediately downstream of the 5′ leader [‘leader-TRS’, reviewed in (5), and studied by transcriptomics for SARS-CoV-2 (6)].

The current COVID-19 pandemic, with its dramatic worldwide impact on human health and economy, is caused by SARS-CoV-2, which emerged in late 2019 in China. The SARS-CoV-2 genome was sequenced in early 2020 (7,8), and currently there are intense worldwide efforts to develop and apply new therapeutic strategies to fight this life-threatening disease. Most of these approaches focus on, first, small-molecule drugs targeting viral enzymes (nucleoside analogs, protease inhibitors and others), and second, on antibodies interfering with virus entry, in particular virus-receptor interactions. In addition, immunomodulatory agents are being used and a large number of SARS-CoV-2 vaccines (including virus vector- and mRNA-based vaccines) are being developed and tested, many of which providing promising new approaches to prevent or treat COVID-19 more effectively (9).

However, alternative novel strategies should also be considered and pursued. Antisense approaches represent such a classical line of sequence-based interference and have been investigated for the last 30 years [for reviews, see (10–13)]. By targeting specific RNA sequences, antisense approaches aim to modulate RNA structure and activity, mRNA splicing, translation, or stability. Best known examples are antisense oligonucleotides (ASO), with incorporated modified positions, such as 2′-*O*-methyl (2′-OMe), 2′-*O*-methoxyethyl (2′-MOE) nucleotides, locked nucleic acids (LNA), morpholino or other modifications, which can increase RNA base-pairing, metabolic stability, and/or delivery. As a result of antisense research over several decades, ASO-based therapies have been advanced to the stage of approved drugs used in certain genetic diseases (14). Regarding antiviral strategies, earlier studies had evaluated HIV-Tat peptide-coupled morpholino ASO against SARS-CoV and the related mouse hepatitis virus (MHV) (15,16). Here, we investigated the antiviral potential of circular RNAs (circRNAs) as a basis for presenting antisense-RNA sequences, exploiting the unusual metabolic stability of circRNAs to develop them into a new line of RNA-based antiviral therapeutics.

CircRNAs are a large class of RNAs with covalently joined 5′ and 3′ ends that exist in all eukaryotes investigated so far and have been known for more than four decades [(17); reviewed in (18–20)]. More recently, circRNAs were rediscovered as a large class of noncoding RNAs, based on deep sequencing (21–23). The most common type of circ-RNAs consists of one or several adjacent exons derived from pre-mRNAs. Biogenesis of exonic circRNAs relies on a kind of alternative splicing (24). Functionally, however, circRNAs have remained largely unexplored, except for a few examples of validated miRNA sponges (23,25), which are embedded in regulatory RNA networks (26,27). Several other hypothetical roles have been proposed for circRNAs, for example protein sponging and antisense activity (28). Based on their unusually high stability, circRNAs provide an attractive basis for constructing designer circRNAs in biotechnological applications [see, for example, (29–31)].

Here, we combined for the first time the classical antisense (AS)-RNA approach with synthetic short circRNAs, integrating antisense sequences into a circRNA backbone. Our overall aim was to interfere with SARS-CoV-2 genome expression and viral proliferation by specifically targeting the structurally conserved 5′-UTR of SARS-CoV-2. Based on structure-guided design and a systematic functional screen of a series of AS-circRNAs, we identified a highly accessible subregion in the SARS-CoV-2 5′-leader that could be efficiently targeted by specific AS-circRNAs, resulting in 90% reduction of viral replication in cell culture. Functional antisense activity was consistently higher when the antisense sequence was presented within a circRNA rather than as a corresponding linear RNA. Compared with 2′-OMe- and 2′-MOE-modified antisense oligonucleotides, unmodified antisense-circRNA was superior in its activity; finally, it was surprisingly robust towards point mutations in the target sequence.

In conclusion, our work establishes AS-circRNAs as a novel molecular approach suitable to target and functionally regulate specific RNAs, therefore opening up promising new avenues to develop highly specific, flexible and efficient therapeutic strategies in molecular virology and medicine.

## MATERIALS AND METHODS

### AS-circRNA design

Antisense target sequences (40–75 nts) were selected based on the SARS-CoV-2 5′-UTR secondary structure (3,4), as well as the presence of specific sequence elements (e.g. 5′-leader, TRS, AUGs). Randomized sequences of equal length (40–75 nts) were used as controls.

RNAs for *in vitro* circularization were composed of a constant backbone sequence, in which the individual antisense target or control sequences were inserted. The constant backbone consists of six complementary nucleotides on either 5′ and 3′ ends of the RNA, followed by four non-complementary nucleotides creating overhanging ends and allowing stem-loop formation and efficient ligation. For enhanced flexibility, and to assure stem loop formation, a spacer of three unrelated nucleotides was added between the constant backbone and the antisense or control sequences on each side (for sequences, see Supplementary Table S1). These sequences were ordered as oligonucleotides (Sigma-Aldrich) including a T7 promoter, and subsequently annealed to yield templates for *in vitro* transcription.

For endogenous overexpression of antisense-circRNAs, oligonucleotide cassettes (see Supplementary Table S1) were cloned into the pAV-U6+27-Tornado-Broccoli vector (32), using the SacII and NotI restriction sites, and replacing the Broccoli aptamer sequence. Again, to allow enhanced flexibility a spacer of unrelated nucleotides was inserted, in this case two nucleotides upstream, and five nucleotides downstream of the AS or control sequence. Since internal poly(U) stretches longer than (U)_4_, including single nucleotide interruptions, would terminate RNA polymerase III, such sequences were changed by single T→A mutations (see Supplementary Table S1).

### *In vitro* transcription, circularization, gel purification and RNase R treatment of antisense-RNAs

RNAs were transcribed from annealed DNA-oligonucleotide templates (see Supplementary Table S1), using the HighScribe™ T7 high-yield RNA synthesis kit (NEB) in the presence of ATP, CTP, UTP, and GTP (each at 7.5 mM), GMP (30 mM GMP; Merck), and RNaseOut (Thermo Fisher Scientific) for 2 h at 37°C. The DNA template was digested by addition of RQ1 DNase (2 U per reaction, Promega), and incubation for 30 min at 37°C. Transcripts were purified using the Monarch RNA purification kit (NEB) and quantified by the Qubit™ RNA broad-range assay kit (Thermo Fisher Scientific).

For circularization, 60 μg transcribed RNA was incubated with 100 U of T4 RNA ligase (Thermo Fisher Scientific) in 1× T4 RNA ligase buffer, supplemented with 0.1 mg/ml BSA and RNaseOut (Thermo Fisher Scientific), overnight at 16°C in a final volume of 200 μl. RNA was recovered by phenol/chloroform extraction (Roth) and ethanol precipitation.

Gel purification was performed as described (33). To validate the circular conformation, 250 ng of gel-purified circular or linear RNA was incubated with or without 2 U of RNase R (Biozym; 30 min at 37°C). After digestion, 200 ng of RNAs were separated in a 10% denaturing polyacrylamide gel and visualized by ethidium bromide staining.

### Luciferase reporter constructs

**Figure 1.**
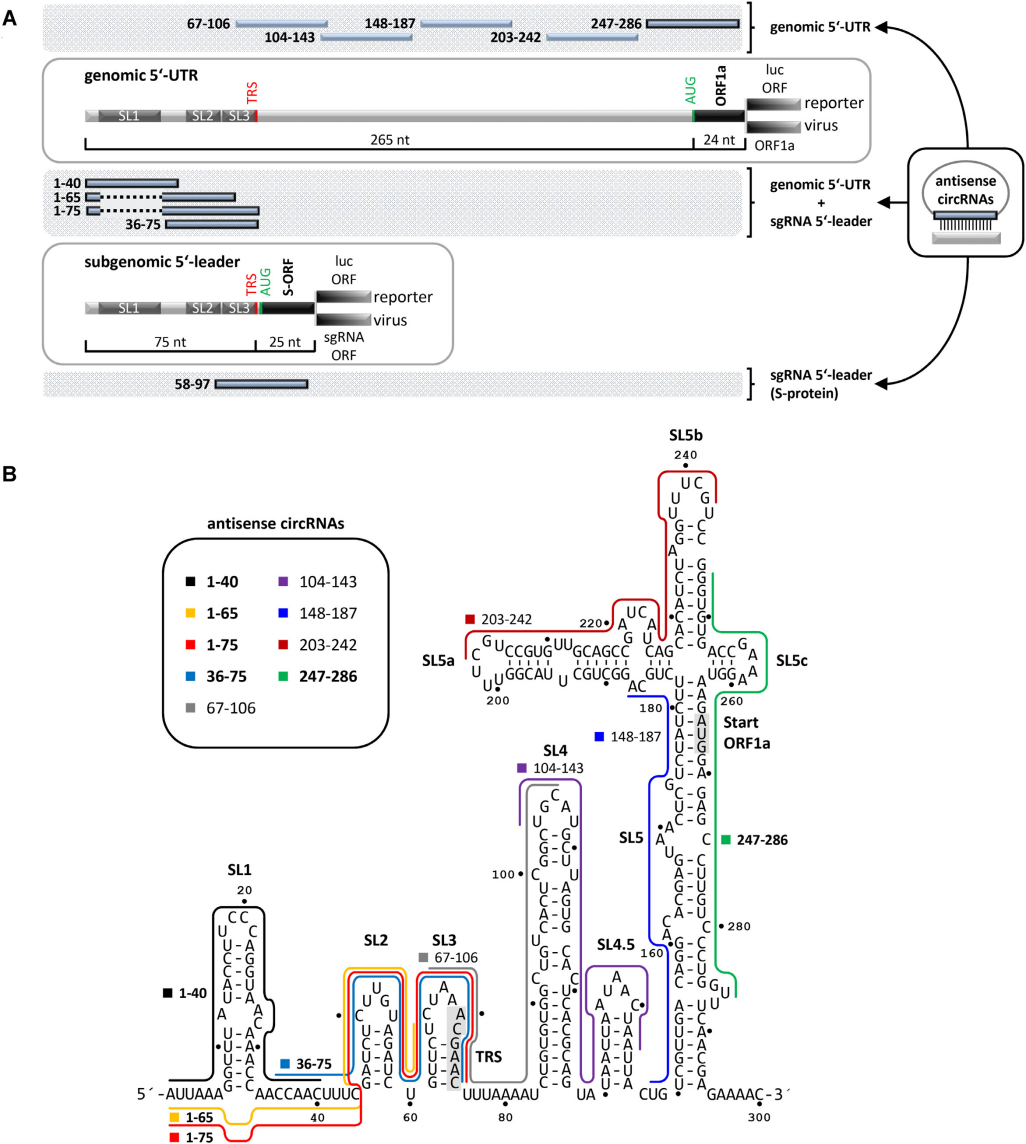
Design of AS-circRNA targeting SARS-CoV-2 RNA. (**A**) Schematic representation of the 5′-UTR (top, nts 1–265) and 5′-leader (bottom; nts 1–75) sequences, targeted by AS-circRNAs, either in a luciferase-reporter (luc), or in the viral SARS-CoV-2 context (ORF1a or S). Target regions of individual AS-circRNAs are represented as blue bars with nucleotide coordinates. The conserved 5′-terminal stem-loop elements (SL1-3) are indicated, as well as the transcription regulatory sequence (TRS) of the 5′-leader. Note that the target regions of AS_1–65 and AS_1–75 circRNAs omit the first stem–loop (SL1), represented as a dashed line. (**B**) Target regions of AS-circRNAs within the 5′-leader (nts 1–75) and 5′-UTR of SARS-CoV-2 (nts 1–265), represented in the context of the secondary structure model of this region [nts 1–300; (3,4)]. For a schematic representation, see panel **A**. The core TRS element (nts 70–75) and the AUG start codon of ORF1a (nts 266–268) are shaded in grey. Note that AS_58–97 circRNA is not included, since it overlaps with the ORF of the S protein.

5′-UTR (nts 1–265) and 5′-leader (nts 1–75) sequences of SARS-CoV-2 (NC_045512.2) were cloned into pcDNA5-CMV-FF (34) containing the Firefly reporter ORF. Additionally, for the 5′-UTR construct, 24 nucleotides of the ORF1a sequence were included, and, for the 5′-leader construct, 25 nucleotides of the S (spike) ORF, followed in either reporter by the Firefly ORF (for a schematic, see Figure 1A). For the construction of 5′-leader constructs carrying point mutations, oligo cassettes with corresponding nucleotide changes were ordered (Sigma-Aldrich) and cloned in front of the Firefly reporter ORF. Point mutations were selected based on their occurrence frequency and annotation within the ViGTK database (https://www.biosino.org/ViGTK).

### Transfection of *in vitro*-transcribed RNAs and Tornado-based circRNA expression constructs; luciferase reporter assays and RT-qPCR

HeLa cells were cultured in DME-medium supplemented with 10% FBS (Gibco) at 37°C and 5% CO_2_. For luciferase reporter assays, 1 × 10^5^ cells were seeded per well (12-well plate). RNA transfections were done using Lipofectamine 2000, and Tornado-plasmid transfections with Turbofect reagent, both in a total volume of 1 ml medium/well (Thermo Fisher Scientific). For AS-circRNA screening, cells were transfected either with 1 μg of individual circRNAs, or with a combination of two circRNAs (0.5 μg of each). For titration experiments, different amounts (100, 250, 500, 750, 1000 ng) of circular or corresponding linear RNAs (CTR2 and 1–75 RNA) were used.

To compare the efficiency of AS-circRNAs and ASOs (2′-OMe or 2′-MOE modified; SeqLab), 1 μg of circRNA and a molar equivalent of the corresponding ASOs were used. Control ASOs carry the same modifications as the ASOs tested (for sequence information, see Supplementary Table S1).

For Tornado-circRNA screening, cells were transfected with 1 μg of plasmid DNA. Culture medium was always changed 1 h prior and 4 h after transfection. After one day, cells were co-transfected with 50 ng of 5′-UTR or 5′-leader luciferase reporter plasmids (see above), together with 5 ng of pRL-SCV40 Renilla-reporter (Promega). At 24 h post-transfection, cells were washed three times with PBS (Gibco), and lysed in 250 μl Lysis-Juice (PJK). Luminescence was measured for Firefly and Renilla luciferase (Beetle- and Renilla-Juice kits, respectively; PJK), using a Centro LB 960 Luminometer (Berthold Technologies). Relative luciferase activities were calculated as a ratio of the Firefly and Renilla raw values, with three technical replicates per sample and a total of three independent biological replicates.

For detection of Firefly mRNA expression levels by RT-qPCR, HeLa cells were seeded and transfected as described above. After one day, cells were co-transfected with 50 ng of 5′-leader luciferase reporter plasmid, together with 5 ng of pRL-SCV40 Renilla-reporter (Promega). 24 h post-transfection, cells were washed three times with PBS (Gibco), and lysed by addition of TRIzol reagent (Thermo Fisher Scientific), and the RNA was purified using the RNeasy kit (Qiagen), followed by DNase digestion with RQ1 DNase (Promega) to remove remaining plasmid DNA. Reverse transcription was performed, using 500 ng total RNA with the qScript cDNA synthesis kit (Quantabio). Real-time qPCR was carried out using the Luna qPCR Reaction Mix (NEB) on a StepOne thermocycler (Thermo Fisher Scientific). Firefly and Renilla reporter mRNAs were amplified by specific primer pairs (see Supplementary Table S1). All reactions were performed in technical and biological triplicates; fold-changes (relative to reporter transfection, but without RNA transfection) were calculated using the ΔΔCt method with average cycle threshold (Ct) values, and Firefly mRNA expression was normalized to Renilla mRNA levels.

### Viral infection, plaque assays and immunofluorescence

Vero E6 cells were cultured in DME-medium supplemented with 10% FBS and 100 U/ml penicillin and 100 µg/ml streptomycin (Gibco) at 37°C and 5% CO_2_. Cells were seeded with a density of 0.5 × 10^5^ per well (24-well plate). RNA transfections were done using Lipofectamine 2000 in a total volume of 0.5 ml medium/well (Thermo Fisher Scientific). For AS-circRNA screening, cells were transfected either with increasing amounts of individual circular RNAs, or their linear counterpart (between 25 and 5000 ng per assay, as described in Results and the figures). Culture medium was exchanged 1 h prior and 4 h after transfection. 24 h post-transfection, cells were inoculated with SARS-CoV-2 at a multiplicity of infection (MOI) of 0.1 pfu/cell at 33°C. At 1 h post-infection, the inoculum was replaced with fresh medium. Virus-containing supernatants were collected at 24 h post-infection, and virus titers were determined by plaque assays (35).

For durability assays (Figure 3E, Supplementary Figure S4), 3 × 10^5^ Vero E6 cells were seeded per well (6-well plate) one day before transfection or infection.

For transfection of RNA prior to viral infection, 5 μg of respective RNAs was transfected, using Lipofectamine 2000 in a total volume of 1 ml medium/well (Thermo Fisher Scientific). The medium was replaced by 2 ml fresh medium after 4 h. 24 h post-transfection, cells were inoculated with SARS-CoV-2 at an MOI of 0.1 pfu/cell at 33°C. At 1 h post-infection, the inoculum was replaced with fresh medium, and cells were subsequently incubated for a total of 72 h.

For transfection post infection, cells were inoculated with SARS-CoV-2 at an MOI of 0.1 pfu/cell at 33°C. At 1 h post-infection, the inoculum was replaced with fresh medium, and 5 μg of respective RNAs was transfected, using Lipofectamine 2000 in a total volume of 1 ml medium per well (Thermo Fisher Scientific). The medium was replaced by 2 ml fresh medium after four hours, followed by incubation of cells for a total of 72 h.

Virus-containing supernatants were collected at 16, 24, 40, 48, 64 and 72 h post-infection or transfection, and virus titers were determined by plaque assays (35).

Cryopreserved normal human bronchial epithelial (NHBE) cells of a non-smoking, healthy donor were obtained from Lonza (CC-2540, Batch No. 18TL269120). The undifferentiated cells were seeded on collagen IV-coated transwell plates (CLS3470-48EA; Corning Costar) and grown in a 1:1 mixture of DMEM (Thermo Fisher Scientific) and BEBM (CC-3171; Lonza), supplemented with BEGM Bronchial Epithelial Single Quots Kits (CC-4175, Lonza) and retinoic acid (74 nM; R2625; Sigma-Aldrich), with medium exchange every second day. After reaching confluence, the cells were cultivated for five (Experiment #1) or seven weeks (Experiment #2) under air–liquid conditions for full differentiation into pseudo-stratified human airway epithelia. The medium from the basolateral compartment was renewed every second day, and the apical surface was washed weekly with PBS (Thermo Fisher Scientific).

The differentiation status of NHBE cells was further validated via immunofluorescence, using antibodies against ZO-1 (Invitrogen, #40-2200, 1:100), mucin 5AC (abcam, #ab198294, 1:100), tubulin IV (abcam, #ab179509, 1:100) and Alexa Fluor 488-coupled F(ab')2 goat anti rabbit IgG (H + L) antibody (Invitrogen, #A-11070, 1:500). Images were acquired by confocal laser-scanning microscopy (Leica TCS SP5), and data were processed, using the Imaris 8.4 software package (Bitplane).

For transfection, the apical surface of the cells was washed three times with 150 μl PBS, and afterwards 5 μg RNA was transfected in a total volume of 125 μl using Lipofectamine 2000 (Thermo Fisher Scientific). Four hours post-transfection, the cells were washed three times with 150 μl PBS. After 24 h the cells were infected with SARS-CoV-2 (MOI of 3 pfu/cell) for 1 h. Afterwards, the inoculum was removed, and at the indicated time points, the apical surface of the cells was incubated with 150 μl/well PBS for 15 min at 33°C, followed by plaque assays to determine virus titers in the supernatants.

### Subcellular fractionation

HeLa cells were seeded with a density of 8 × 10^5^ cells per 6-cm plate and transfected with 3 μg of *in vitro* produced linear or circular RNA or with 4 μg of Tornado-plasmids, using Lipofectamine 2000 in a total volume of 4 ml medium/plate. After 24 h, cells were harvested, and 2 × 10^6^ cells subjected to fractionation, using the NE-PER Nuclear and Cytoplasmic Extraction kit (Thermo Fisher Scientific). RNA for Northern blot analysis was prepared from 75% of the nuclear and cytoplasmic fractions using TRIzol LS (Ambion), while 25% was saved for Western blotting (see below).

### Western blot

Viral protein accumulation was analyzed by Western blot of Vero E6 cell lysates, previously transfected with 2.5 μg of circRNAs and infected with SARS-CoV-2 at an MOI of 0.1 pfu/cell at 33°C. Total protein lysates obtained at 24 h post-infection were heat-denatured in SDS-loading buffer (50 mM Tris–HCl pH 6.8, 2% SDS, 10% glycerol, 2.5% 2-mercaptoethanol, and 0.05% bromophenol blue) at 95°C for 10 min. Following separation by SDS-polyacrylamide gel electrophoresis (PAGE; 10%), proteins were blotted onto a nitrocellulose membrane (BioRad). Viral proteins were immunostained overnight with rabbit anti-SARS nucleocapsid protein (N, 200-401-A50; Rockland Immunochemicals, 1:500) or mouse anti-GAPDH antibody (monoclonal antibody G8795, GAPDH-71.1; Sigma-Aldrich, 1:5000) and appropriate secondary antibodies (HRP-conjugated anti-rabbit (A0545-1ML) or anti-mouse (A9044-2ML), Sigma-Aldrich, each 1:10,000). The blots were developed using the Lumi-Light Western-Blotting Substrate (Roche). Western blot signals were estimated by densitometry, using GelAnalyzer 19.1 software.

For subcellular fractionation, 1.25% of cytoplasmic or nuclear fractions were analyzed by SDS-PAGE (10%) and Western blotting through detection of hnRNP A1 (monoclonal antibody, sc-32301, 4B10; Santa Cruz Biotechnology, 1:2000) and GAPDH (monoclonal antibody G8795, GAPDH-71.1; Sigma-Aldrich,1:5000).

### Northern blot

All Northern blots were performed as previously described (36).

#### Denaturing polyacrylamide Northern blot

For detection of *in vitro* produced linear and circular RNAs transfected in HeLa or Vero-E6 cells, 1 μg of total RNA or 20% of cytoplasmic/nuclear fractions was used, and for Tornado-derived circRNAs 250 ng of total RNA or 20% of cytoplasmic/nuclear fraction. Samples were separated on a 10% denaturing polyacrylamide gel, transferred to a nylon membrane (Hybond-N+; Amersham) by semi-dry blotting, and crosslinked by UV light (0.125 mJ/cm^2^ at 254 nm). Membranes were subsequently hybridized with DIG-UTP-labeled (DIG RNA Labeling Mix, Roche) riboprobes in NorthernMax hybridization buffer (Thermo Fisher Scientific) at 60°C. For *in vitro* produced RNAs, CTR2 or 1–75 specific riboprobes were used that are able to detect both circular and linear molecules. For detection of Tornado circRNAs, a circular-junction-specific probe was used. For oligonucleotide and riboprobe sequences, see Supplementary Table S1. Probe detection with alkaline phosphatase-conjugated anti-DIG-Fab fragments (11093274910, Roche) and CDP-Star chemiluminescence substrate was done as described (Roche).

#### RNase R and RNase H treatment

To confirm circularity of the detected Tornado-derived circRNAs, 250 ng of total RNA were either incubated with 5 U/μg RNase R (Biozym) in 1x RNase R buffer for 1 h at 37°C, or with 50 ng of antisense DNA-oligonucleotide in 1x RNase H buffer for 20 min at 37°C, followed by addition of 1 U RNase H (NEB) and incubation for 40 min at 37°C. To assess whether the effect of AS-circRNAs is based on a blocking or cleavage mechanism, 3 μg of total RNA from previously transfected HeLa cells (5 μg circRNA, 500 ng 5′-leader reporter) were incubated with 600 ng antisense DNA-oligonucleotide and RNase H-treated (see above). Samples were analyzed by denaturing polyacrylamide Northern blot as described above.

#### Glyoxal Northern blot

For detection of the SARS-CoV-2 genome and subgenomic RNAs, 3 μg of total RNA from previously transfected (5 μg *in vitro* produced linear or circular RNAs per 3 × 10^5^ cells on a 6-well plate, in 1 ml total volume), and infected (at 24 h post-infection; MOI = 0.1 pfu/cell) Vero E6 cells were mixed with glyoxal loading buffer (Ambion) and incubated for 30 min at 50°C. RNAs were separated by 1% agarose gel electrophoresis in 1x MOPS buffer, transferred to a nylon membrane (Hybond-N+, Amersham), and hybridized with a DIG-labelled riboprobe complementary to the 3′-UTR common to the positive-strand viral genome as well as the subgenomic RNAs (SARS-CoV-2 genome positions 29,535 to 29,848; for the probe sequence, see Supplementary Table S1; NorthernMax hybridization buffer; Thermo Fisher Scientific; 68°C). The same procedure was used for detection of the full-length 5′-leader reporter transcript: 3 μg of total RNA from previously transfected HeLa cells (5 μg circRNA, 500 ng 5′-leader reporter) were treated and separated as described above, followed by probing with a DIG-labeled riboprobe (position 1 to 997 of the reporter-derived transcript, see Supplementary Table S1). Probe detection was performed as described above.

### RNA-Seq and data analysis

For global analysis of viral genome and subgenomic RNA abundance after transfection of *in vitro* produced linear or circular RNA, 3 × 10^6^ Vero E6 cells were seeded in a 6-well plate 1 day before transfection. 5 μg of respective *in vitro* produced RNA was transfected in 1 ml total volume, using Lipofectamine 2000. At 24 h post-transfection, cells were inoculated with SARS-CoV-2 at an MOI of 0.1 pfu/cell at 33°C. At 24 h post-infection, RNA was isolated by TRIzol extraction (Ambion). 1 μg of total RNA, together with 2 μl of a 1:100 dilution of ERCC standard (Ambion) as spike-in, was used to perform poly(A)-selection (NEBNext^®^ Poly(A) mRNA Magnetic Isolation Module; NEB), followed by library preparation (NEBNext Ultra II Directional RNA Library Prep Kit for Illumina; NEB). Libraries were sequenced on an Illumina NextSeq 500 platform (single-read, 150 bp). RNA-seq data were deposited in the Sequence Read Archive (PRJNA693241) of NCBI. Sequence reads were aligned to the SARS-CoV-2 reference genome sequence (NC_045512.2) using STAR (37). Positions of the transcription regulatory sequences (TRS) and subgenomic RNAs (sgRNAs) were based on the SARS-CoV-2 transcriptome tracks (6).

To quantitate the total viral RNA accumulation in each sample (Figure 4B), and to calculate the read coverage in the nine segments across the SARS-CoV-2 viral genome (Figure 4C), the number of uniquely mapped sequence reads of each sample were normalized with the corresponding number of total sequenced reads (mock: 33.4 mio; CTR2_lin: 49.5 mio; CTR2_circ: 66.4 mio; 1–75_lin: 46.5 mio; 1–75_circ: 40.1 mio). For the read coverage across the viral genome, the terminal regions were not used (positions 1–75 and the 3′-terminal 50 nucleotides), due to their low representation.

## RESULTS

### Design of AS-circRNAs targeting SARS-CoV-2 RNA

To develop new RNA-based therapeutics for antiviral strategies, we designed and tested artificial small circRNAs containing antisense-RNA sequences that target SARS-CoV-2 RNA. We focussed on the 5′-UTR, because its RNA secondary structure is relatively well characterized and highly conserved (2,3), and there is evidence for important functions of the 5′-UTR on multiple levels, including viral genome replication (38) and transcription [subgenomic RNA (sgRNA) synthesis, (5,6)], translational initiation (39), RNA stability (3) and, potentially, RNA packaging (40). To screen for functional antisense sequences and optimal SARS-CoV-2 targets in the 5′-UTR, we initially used two separate luciferase reporter systems (Figure 1A).

First, to assess effects of antisense-circRNAs (AS-circRNAs) on the 5′-UTR of the viral genome, the first 265 nucleotides of the SARS-CoV-2 genome including the ORF1a translation start codon and the first 24 nucleotides of ORF1a, were fused in-frame with the luciferase ORF, resulting in the ‘5′-UTR’ reporter construct (Figure 1A).

Second, to determine effects on the 5′-UTR of viral sgRNAs, the 5′-terminal 75 nucleotides of the SARS-CoV-2 genome were used, comprising the common ‘leader’ sequence (including the TRS element), which is present on all sgRNAs. That region, followed by 25 nucleotides with AUG start codon and 5′-terminal coding sequence of the S protein, were fused to the luciferase ORF, resulting in the ‘5′-leader’ reporter (Figure 1A).

We selected the exact positioning of the antisense sequences according to the current secondary structure model of the 5′-UTR and 5′-leader regions, containing three highly conserved stem-loop RNA structures [SL 1–3, (3,4)]. Our previous work on AS-circRNAs had indicated that perfect base-pairing between circRNA and target over at least 30 nucleotides was required for stable interaction (Silke Schreiner and Christina Pfafenrot, unpublished observations).

On this basis, a series of six short AS-circRNAs, between 66 and 76 nts in length and with 40–50 nts of antisense sequence, was designed to specifically target the SARS-CoV-2 5′-UTR regions (named according to target boundaries; for a schematic representation and exact SARS-CoV-2 target boundaries, see Figure 1A and B; antisense sequences are listed in Supplementary Table S1). AS-circRNA 1–40 targets the 5′-terminal 40 nucleotides of the SARS-CoV-2 genome (and sgRNAs), including SL1; AS-circRNA 1–65 targets the 5′-terminal 65 nucleotides, but omits the complete 27-nucleotide SL1, and AS-circRNA 1–75 extends this base-pairing interaction by ten nucleotides, including the TRS element. AS-circRNA 36–75 again was designed to base-pair with the single-stranded region between SL1 and 2, and the less stable SLs 2 and 3, including the TRS element. These four AS-circRNAs have in common that they can base-pair with both the SARS-CoV-2 genomic RNA and all eight major sgRNAs (sgRNAs 2–9) produced in infected cells.

In addition, two AS-circRNAs were designed to specifically target either the ORF1a translation start site, along with flanking regions (in the SARS-CoV-2 genome RNA), or the ORF encoding the viral S protein (in sgRNA 2): AS-circRNA 247–286 spans the 3′-terminal region of the genomic 5′-UTR region and the first 21 nucleotides of the ORF1a, whereas AS-circRNA 58–97 targets SL3, the TRS element and the first 21 nucleotides of the S-protein ORF (Figure 1A). As control and for normalization of luciferase activities, two non-specific circRNAs were used in these experiments, each comprising a randomized sequence of 40 nucleotides instead of the antisense sequences.

### Synthetic AS-circRNAs inhibit translation of SARS-CoV-2 reporter constructs

We initially used AS-circRNAs transiently overexpressed in HeLa cells by the so-called Tornado system [Twister-Optimized RNA for Durable Overexpression, see (32)] which relies on an RNA polymerase III-driven and self-cleaving expression cassette, combined with circularization by endogenous RtcB tRNA ligase (Supplementary Figure S1A). One day after transfection of the circRNA expression constructs, either genomic 5′-UTR or subgenomic 5′-leader reporters were transfected, followed by luciferase assays (Supplementary Figure S1B). Overexpression at similarly high levels of all ten SARS-CoV-2 5′-UTR/5′-leader specific AS-circRNAs, as well as of two control circRNAs, was confirmed by Northern blot analysis (Supplementary Figure S1C). In addition, circular configuration was stringently established by RNase H cleavage assays (for AS_1–75 circRNA), and cellular distribution between nucleus and cytoplasm was characterized (for AS_1–75 and CTR2 circRNAs, see Supplementary Figure S1DE). Based on reporter assays with both the genomic (5′-UTR) and subgenomic (5′-leader) constructs, all of these overexpressed anti-SARS-CoV-2 circRNAs, except for AS_67–106 and AS_104–143, were found to reduce luciferase expression to levels between 50 to 60% (Supplementary Figure S1F). Since the four AS-circRNAs against the 5′-leader region (nucleotides 1–75) as well as three AS-circRNAs upstream of or spanning the ORF1a translation initiation site (nucleotides 148–286) reduced reporter expression most profoundly, we decided to focus our subsequent analysis on these regions.

However, the Tornado-based circRNA expression system results in massive overexpression, in the order of 10^6^ copies per cell (31), and one cannot rule out effects due to linear precursors or side-products. Therefore we switched for all following assays to synthetic AS-circRNAs transfected in mammalian cells, which is advantageous, because it is a biochemically well-defined system; for example, transfected circRNA quantities can be titrated and the effects of circular and linear forms can be directly compared with each other.

**Figure 2.**
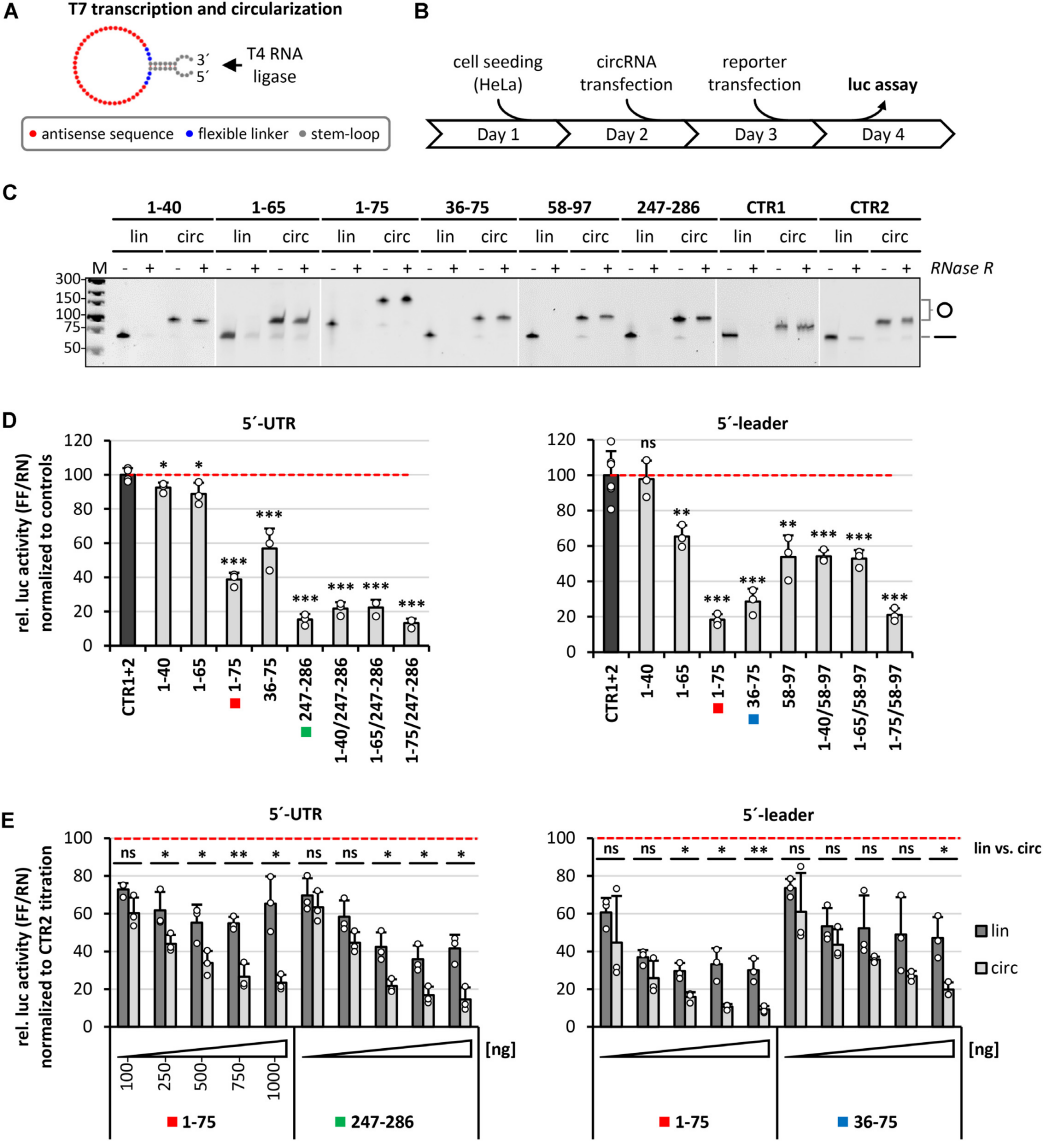
Screening of active AS-circRNAs: 5′-UTR and 5′-leader reporter assays. (**A**) Design of synthetic AS-circRNAs. CircRNAs were produced by *in vitro* T7 transcription and subsequent circularization by T4 RNA ligase. Each circular RNA is composed of a stem–loop with overhangs for efficient ligation (grey), a short stretch of unrelated nucleotides for enhanced flexibility (blue), and the antisense sequence (red). (**B**) Experimental workflow for luciferase reporter assays in HeLa cells transfected with synthetic circRNAs. (**C**) AS-circRNA synthesis. RNase R treatment and aberrant electrophoretic migration confirm the circularity of the produced circRNAs. Gel-purified linear and circular RNAs (lin/circ) were treated with RNase R, or left untreated (–/+), and analyzed by denaturing polyacrylamide electrophoresis and ethidium bromide staining. Mobilities of circular (o) and linear (–) forms are marked. *M*, DNA markers (sizes in bp). (**D**) Luciferase reporter assays reveal regions accessible to AS-circRNAs, based on the repression of luciferase activity by specific SARS-CoV-2 5′-UTR (left) and 5′-leader reporter constructs (right). HeLa cells were transfected with the respective circRNA (as indicated below the diagram) or a combination thereof (e.g. AS_1–40/247–286). The color code highlights those AS-circRNAs that were analyzed in more detail in panel E. After 24 h, the respective reporter was transfected (5′-UTR or 5′-leader), and relative luciferase activities (ratio of Firefly and Renilla expression) were measured, normalized to control circRNAs CTR1 and 2 (mean and standard deviations of three replicates, *P < 0.05, **P < 0.005, ***P < 0.001, ns = not significant, two-sided *t*-test). **(E)** Dose dependence and comparison of circular versus linear configuration of selected antisense-RNA regions. HeLa cells were transfected with increasing amounts (100–1000 ng per assay) of circRNAs (light gray), or their linear counterparts (dark grey; as indicated below the diagram). After 24 h, the respective reporter constructs were transfected (5′-UTR or 5′-leader), and relative luciferase activities (Firefly/Renilla expression ratios) were measured, normalized to control circRNA CTR2 (mean and standard deviations of three replicates, *P < 0.05, **P < 0.005, ***P < 0.001, ns = not significant, two-sided *t*-test).

AS-circRNAs were produced *in vitro*, based on transcription by T7 RNA polymerase and circularization by T4 RNA ligase, followed by gel purification (Figure 2A). Each circRNA was designed such that the antisense sequence was linked to a common short stem–loop (6 perfect base-pairs with two overhanging ends that were joined by ligation). The antisense region and the stem–loop are connected by two three-nucleotide linkers, to allow a more flexible presentation of the antisense sequence.

To functionally characterize this series of AS-circRNAs, we first tested their ability to inhibit translation in the two luciferase reporter systems (for a flowchart of analysis, see Figure 2B): HeLa cells were first transfected with circRNA; after 24 h, the reporter construct was transfected and, after another 24 h, luciferase activities were measured.

For quality control and evidence of circularity, purified AS-circRNAs (as well as their linear counterparts) were characterized by denaturing PAGE (Figure 2C): Note that all of these relatively short circRNAs are RNase R resistant, in contrast to the corresponding linear RNAs, and that the circular configuration results in slower mobility relative to the linear form, demonstrating circularity. Regarding cellular distribution after transfection, AS-circRNAs as well as their linear counterparts were detected predominantly in the cytoplasmic compartment (where coronavirus replication is localized), as shown for AS_1–75 and control linear and circular RNAs (Supplementary Figure S2).

Comparing the two reporters, 5′-UTR versus 5′-leader, corresponding effects of AS-circRNAs were observed for the first four AS-circRNAs that target the 5′-terminal region, nucleotides 1–75 (AS_1–40, AS_1–65, AS_1–75, and AS_36–75; see Figure 2D): Only in case of the longest version, AS_1–75, which excludes stem-loop 1, both reporters were strongly reduced in translation (to 39 and 18% residual level, respectively). The two shorter AS-circRNAs (AS_1–40 and AS_1–65) showed smaller or insignificant activities. The strong effect of AS_1–75 circRNA is not simply due to extended base-pairing, since the shorter AS_36–75 was almost as strong as AS_1–75: reduction to 57 and 29%, respectively, for the two reporters.

Moving from the 5′-end to the regions overlapping the translation start codons for the ORF1a and the S-gene, we assayed AS_247–286 circRNA (for the genomic 5′-UTR reporter) and AS_58–97 circRNA (for the 5′-leader reporter): Both had strong effects on reporter activity (reduction to 15% and 54%, respectively). The use of AS-circRNAs (AS_247–286 and AS_58–97) targeting the AUG start codon regions (of ORF1a and S-gene, respectively) in combination with circRNAs targeting the 5′-terminal leader region did not further increase the inhibitory action on reporter translation (see three combinations for each of the two reporters, Figure 2D).

To address the important question of whether the circular configuration of the AS-circRNA is important for inhibitory activity, we compared the two AS-circRNAs with the strongest inhibitory effects with their linear counterparts: AS_1–75 and AS_247–286, using the 5′-UTR reporter, and AS_1–75 and AS_36–75, using the 5′-leader reporter (Figure 2E). To provide additional support for the specificity of the inhibition, we also measured dosis-dependent reduction in reporter activity for the selected RNAs, with doses ranging between 100 and 1000 ng per assay. For each of the four setups, we observed clear dosis-dependent effects, in particular for the circular configuration. Under the conditions used, maximal activities were attained with 750–1000 ng of AS-circRNA per assay, whereas the inhibition with linear counterparts leveled off at 500 ng and above. Importantly, the circRNAs were consistently more potent than their linear counterparts. At higher concentrations, the circRNA caused a more than 2-fold stronger reduction of reporter activity than the corresponding linear RNA. This superior inhibitory efficacy of the AS-circRNAs may be due to differential stabilities or intracellular localizations of the transfected RNAs, their intrinsic antisense activity, base-pairing potential, structural properties, or a combination thereof.

We conclude that, based on two separate reporters and AS-circRNA transfection in HeLa cells, both the 5′-leader- and the AUG-start codon-proximal regions can be efficiently targeted by AS-circRNAs, resulting in strong translational inhibition down to 10% residual level.

### Inhibition of SARS-CoV-2 proliferation by designer AS-circRNAs

**Figure 3.**
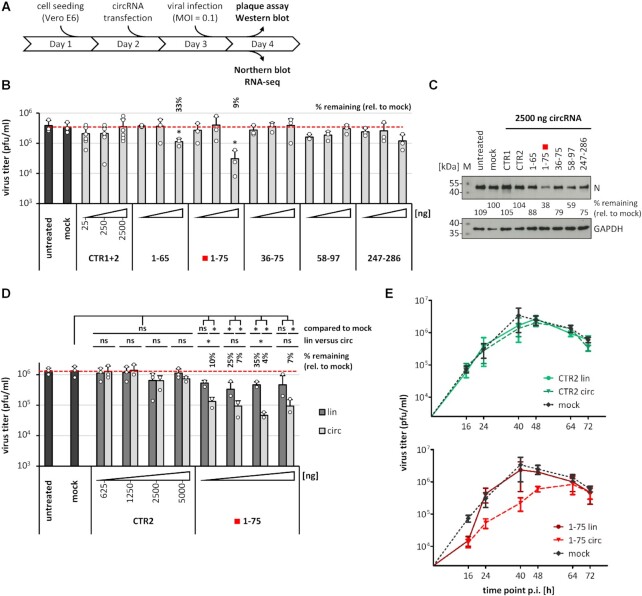
Inhibition of SARS-CoV-2 proliferation by AS-circRNAs: viral infection assays. (**A**) Experimental workflow for viral infection assays using Vero E6 cells. (**B**) Screening of AS-circRNAs by virus titer assays identifies AS_1–75 as the most effective antiviral circRNA. Vero E6 cells were transfected with increasing quantities of circRNAs (25, 250 and 2500 ng per assay; light gray; as indicated below the diagram). After 24 h, cells were infected with SARS-CoV-2 (MOI = 0.1 pfu/cell). The effects on virus titers were measured by virus plaque assays using cell cutlure supernatants collected at 24 h post-infection (mean and standard deviations of three experiments, *P < 0.05, ns = not significant, two-sided *t*-test). Untreated (without RNA and transfection reagent) and mock-treated cells (without RNA, but with transfection reagent) served as controls. Residual virus titers of significantly affected samples are indicated as ‘percent remaining’ relative to mock treatment. (**C**) Viral protein synthesis assays: Western blot analysis of the viral nucleocapsid protein (N) confirms reduction of viral protein accumulation in cells treated with specific AS-circRNAs. Vero E6 cells transfected with 2500 ng of respective circRNAs per assay were harvested, lysed, and equal amounts of protein were analyzed by Western blotting, using the nucleocapsid protein as a marker for viral protein accumulation (quantitation of protein levels relative to mock); GAPDH was used as loading control. *M*, protein markers (sizes in kDa). (**D**) AS_1–75 circRNA: dose dependence of antiviral effect, in comparison to its linear counterpart. Vero E6 cells were transfected with increasing amounts (625, 1250, 2500 and 5000 ng) of AS_1–75 circRNA (light gray), or of its linear counterpart (dark gray), followed by viral infection (MOI = 0.1 pfu/cell) after 24 h. Plaque assays were used to determine virus titers in culture supernatants collected at 24 h post-infection (mean and standard deviations of three experiments, *P < 0.05, ns = not significant, two-sided *t*-test). As controls, untreated (without RNA and transfection reagent) and mock-treated cells (without RNA, but with transfection reagent) were used, as well as transfections with linear or circular control RNA (CTR2). The virus titer of significantly affected samples is indicated as ‘percent remaining’, relative to mock treatment. (**E**) Durability of antiviral activity of AS_1–75 circRNA. Vero E6 cells were transfected with AS_1–75 circRNA or its linear counterpart (bottom panel; in red), followed by viral infection (MOI = 0.1 pfu/cell) after 24 h (mean and SEM of three experiments). Plaque assays were performed to determine virus titers in culture supernatants collected at the indicated time points (16–72 h post-infection). As controls, mock-treated cells (without RNA, but with transfection reagent) were used (top and bottom panels; in black), as well as transfections with linear or circular control RNA (CTR2; top panel; in green).

Following the identification of specific AS-circRNAs that effectively inhibit translation of reporter RNAs, we sought to corroborate key findings of our study by using SARS-CoV-2-infected cells. To this end, the set of AS-circRNAs we had characterized in our reporter assays, except for inactive AS_1–40, as well as two control RNAs, were transfected in Vero E6 cells, which are permissible for SARS-CoV-2 infection and allow the production of infectious virus progeny (for a flowchart, see Figure 3A). Each AS-circRNAs was transfected in three different quantities (25, 250 and 2500 ng per assay), and 24 h later, cells were infected with SARS-CoV-2 at an MOI of 0.1 pfu/cell. 24 h post-infection, cell culture supernatants were collected, and virus titers were determined by plaque assays to assess the antiviral effects of individual AS-circRNAs on viral replication in cell culture (Figure 3B).

Compared to the controls (untreated cells, mock-transfected cells, two different control AS-circRNAs), the three AS-circRNAs targeting the untranslated leader region differentially affected virus titers: The strongest effect on viral proliferation was measured for AS_1–75, a reduction to 9% of control level, consistent with the strong effect observed in the previous reporter assays (compare Figures 3B and 2D). The shortened version of this circRNA, AS_1–65, still had a moderate, but significant effect on virus titers (reduction to 33% compared to untreated control cells), whereas AS_36–75 had no significant effect (Figure 3B). In contrast to the initial reporter assays, AS_58–97, designed as specific for the subgenomic mRNA encoding the S protein, and AS_247–286, targeting the genomic region including the ORF1a start codon, did not significantly reduce virus titers (Figure 3B). Based on these results, we focussed our further analysis on the AS_1–75 circRNA, which targets both viral genomic and all subgenomic RNA species.

To obtain additional evidence for inhibition of viral replication, we also measured viral protein synthesis by Western blotting, using SARS-CoV-2 nucleocapsid (N) protein-specific antibodies (Figure 3C). Clearly, compared to the controls (untreated, mock, CTR 1 and 2) and four other AS-circRNAs, intracellular accumulation of viral N protein was most profoundly reduced in the cells treated with AS_1–75 circRNA, consistent with the observed inhibitory effects of this particular circRNA on the production of infectious virus progeny.

Finally, we compared effects of circular and linear versions of the antisense sequence of AS_1–75 in their efficiency on viral proliferation, using an extended range of RNA quantities (625–5000 ng per assay; see Figure 3D). We observed a strong and dosis-dependent antiviral effect of AS_1–75 circRNA, with virus titers reduced down to 4% (at 2500 ng per assay); at an even higher dose (5000 ng), virus titers were reduced to 7%, perhaps reflecting some toxic effects at this high circRNA quantity or suboptimal ratios of circRNA to transfection reagent. In contrast, control AS-circRNA had no significant effect. Comparing the effects of circular versus linear RNAs, we observed at all quantities assayed, that circRNA was clearly superior to the corresponding linear version, consistent with our results from reporter assays (see above and Figure 2E).

In addition to the general workflow (Figure 3A), in which we monitored the increase of virus titers until 24 h post-infection, we addressed the question of whether the antiviral effect of AS_1–75 circRNA persists for longer time periods (Figure 3E): We determined virus titers in the culture supernatants of cells transfected with linear versus circular AS_1–75 RNA (along with appropriate controls) and collected at six time points from 16 to 72 h post-infection. Whereas effects of linear and circular control RNA (CTR2) did not significantly differ from the mock control, AS_1–75 circRNA caused a significant reduction of virus titers, especially between 24 to 48 h post-infection. This antiviral effect was specific for the circular configuration and indicates durability of the effect over at least two days under the conditions used. Finally, combining virus titer measurements from several assays of the antiviral effect of AS_1–75 circRNA (all measured at 24 h post-infection and based on Figures 3B, D, E), we estimated an EC_50_ value in the 20–50 nM range for this most effective AS-circRNA (Supplementary Figure S3).

Furthermore, to answer the question whether AS_1–75 circRNA also shows an effect in cells already infected with virus, additional experiments were performed in reverse order of circRNA transfection and virus infection (see Supplementary Figure S4): One hour after virus infection, linear or circular AS_1–75 RNA (together with appropriate controls) was transfected, and virus titers were determined in the culture supernatants at six time points after RNA transfection, from 16 to 72 h post-infection. We chose a one-hour interval between virus infection and circRNA transfection, as used in many published studies. Binding and uptake of virus particles is a relatively fast process (for example, see reference 41). Therefore, one hour is sufficient for establishment of the viral infection, and at the same time, we avoid any potential problems with dying cells, which at later time points would accumulate, obscure any effects, and make interpretations difficult. Again, in this reverse order, the effects of linear and circular control RNA (CTR2) were not significantly different from the mock control, whereas AS_1–75 circRNA caused a significant reduction in viral titers, especially between 24 and 64 h post-transfection. The antiviral effect was also more pronounced for the circular than for the linear configuration, as observed in the usual setup (Figure 3E).

In addition to the standard Vero E6 cell culture system, we evaluated the antiviral effect of AS_1–75 circRNA in a biologically more relevant, *ex-vivo* respiratory cell culture system, based on differentiated primary normal human bronchial epithelial (NHBE) cells grown in air/liquid interface culture (Supplementary Figure S5). This mimicks the tracheobronchial region of the human respiratory tract in a physiologically relevant cellular environment (42). Differentiated NHBE cells were transfected with linear versus circular AS_1–75 RNA or CTR2, followed by viral infection with SARS-CoV-2 after 24 h and determination of virus titers in the culture supernatants at different time points (24–72 h post-infection). Comparing two independent experiments, we conclude that also in this *ex-vivo* model system AS_1–75 circRNA (as well as its linear counterpart) exhibits a strong inhibiting effect on viral replication.

To assess if an elongation of the antisense region of AS_1–75 further improves its antiviral activity, we tested two more AS-circRNAs in virus titer assays: AS_1–100 (S) and (N), both increasing the base-pairing potential of AS_1–75 circRNA by 25 positions and including a small number of nucleotides from the 5′-terminal coding regions of the S and N genes, respectively (Supplementary Figure S6). However, compared to AS_1–75 (virus titer reduced to 1%, relative to control), both elongated AS-circRNAs were slightly less active (reduction to 7 and 4%, respectively, relative to the CTR3 control circRNA of equal length). Note that the elongated AS-circRNAs were designed to specifically target the sgRNAs that are used for S- and N-protein expression, respectively, whereas AS_1–75 has a broader specificity by targeting both genomic RNA and all plus-strand subgenomic RNAs.

**Figure 4.**
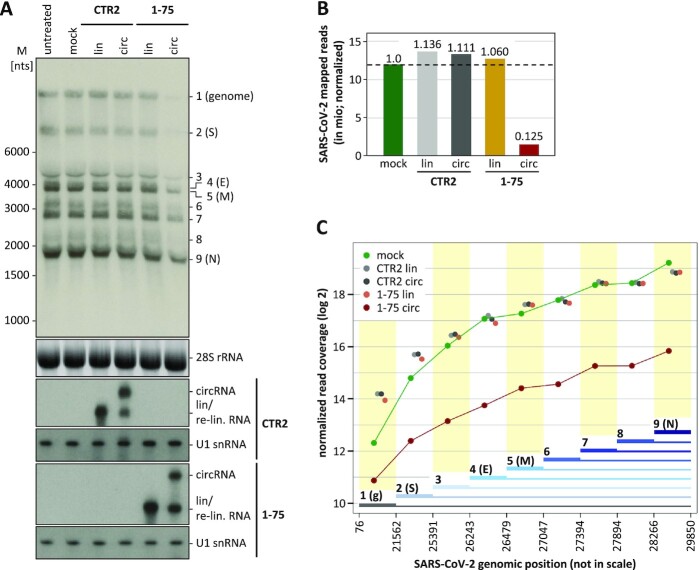
Inhibition of viral RNA synthesis and processing by AS_1–75 circRNA. (**A**) Northern blot analysis of genomic and subgenomic viral RNAs. Vero E6 cells were transfected with AS_1–75 circRNA or its linear counterpart; as controls, untreated (without RNA and transfection reagent) and mock-treated cells (without RNA, but with transfection reagent) were used, as well as cells transfected with linear or circular control RNA (CTR2). At 24 h post-transfection, cells were infected with SARS-CoV-2 (MOI = 0.1 pfu/cell). At 24 h post-infection, total RNA was prepared and subjected to glyoxal-Northern blot analysis, to detect genomic and all subgenomic viral RNA species. *M*, RiboRuler High Range RNA Ladder (Thermo Fisher Scientific). As input control, 28S rRNA was detected by ethidium bromide staining, and CTR2 and AS_1–75 RNAs by specific Northern probes. (**B**) RNA-seq analysis of total viral RNA synthesis. The total numbers of SARS-CoV-2-mapped reads (in mio; normalized to total read number) were compared for RNAs isolated from virus-infected Vero E6 cells that were mock-transfected or transfected with CTR2 control and AS_1–75 RNA, each in linear or circular form, with ratios of read numbers relative to mock conditions indicated. (**C**) Effect of AS_1–75 circRNA on viral genome (g) and subgenomic RNA production in infected cells. Cumulative read coverages (in log_2_; normalized to total read number) are plotted for mock-treated, and CTR2 control RNA (lin/circ) or AS_1–75 RNA (lin/circ)-transfected cells. The SARS-CoV-2 genome was divided in nine sections with boundaries defined by the body-TRS sites of the eight subgenomic RNAs (sections used for cumulative read numbers are marked by thick lines; genomic positions are indicated below and drawn not in scale).

Finally, we assessed the effect of AS_1–75 RNA on viral RNA synthesis more directly (Figure 4). Viral genome RNA and all subgenomic mRNAs were detected in infected and transfected cells by Northern blotting with a probe specific for the 3′-terminus of the genome (Figure 4A). We compared the effects of linear and circular versions of AS_1–75 with four different controls (untreated cells, mock, linear and circular control CTR2 RNA). Only the circular configuration of AS_1–75, but not its linear version, strongly reduced viral RNA levels, both genomic RNA and all subgenomic RNAs that can be resolved, indicating a global effect of this most active AS-circRNA on viral RNA synthesis.

Since Northern blotting cannot be quantitated precisely under these conditions, and not all eight subgenomic RNA species (RNAs 2–9) could be unambiguously identified, we performed RNA-seq, using poly(A)-selected RNA from infected cells. By comparing normalized SARS-CoV-2 genome-mapped reads, which reflect total viral RNA accumulation in infected cells, we found that only the read-counts obtained for cells transfected with AS_1–75 circRNA were strongly reduced (to 12.5% of mock levels; Figure 4B).

In order to analyze individual viral RNA species, we measured the read coverage for nine segments across the SARS-CoV-2 viral genome, each of which are delimited by flanking TRS sites (Figure 4C). Due to the characteristic 3′-coterminal structure of each of the viral RNA species, only full-length viral genome RNA (g) can be assessed directly by read coverage between positions 76 and 21,562. In contrast, production of the individual subgenomic RNAs can be determined only as ‘cumulative read coverage’: For example, the read coverage between positions 21,562 and 25,391 combines reads for both the genome RNA (RNA 1) and the sgRNA 2 (S).

When we compared cumulative read coverage across the viral genome sequence for cells transfected with linear AS_1–75 RNA, AS_1–75 circRNA, or one of the controls, we found that only in the AS_1–75 circRNA-transfected cells viral RNA levels were strongly reduced (to between 37 and 10% relative to mock). Moreover, the extent of reduction increased with a 5′-to-3′ gradient, and most profoundly within the first three segments, proceeding from viral genome RNA to regions that include more and more sgRNAs. This characteristic behavior suggests that the AS_1–75 circRNA interferes, at least in part, with sgRNA synthesis.

### AS-circRNAs exhibit robust activity against SARS-CoV-2 mutant sequences and are superior to modified ASOs

**Figure 5.**
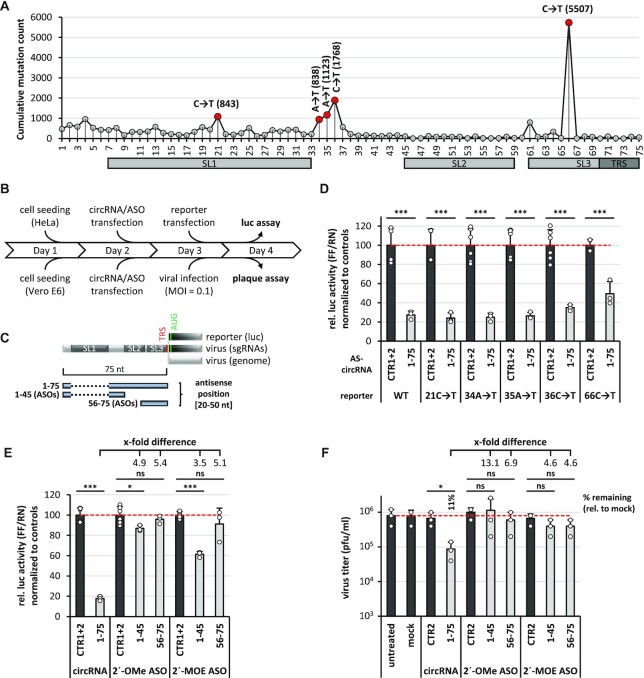
AS-circRNAs exhibit robust activity against SARS-CoV-2 mutant sequences and are superior to modified ASOs. (**A**) Summary of naturally occurring point mutations within the viral 5′-leader of SARS-CoV-2. All annotated mutations are indicated per nucleotide position [cumulative mutation count, as deposited in the ViGTK database (https://www.biosino.org/ViGTK/); as at 30 April 2021). The five most frequent single-point mutations in the 5′-leader region (positions 1–75) are highlighted in red (occurrences given in parentheses) and were selected for mutational analysis. Secondary structures and regulatory elements are marked (SL1–3, TRS). (**B**) Experimental workflow for luciferase reporter assays in HeLa cells, and for viral infection assays using Vero E6 cells, transfected with synthetic circRNAs or modified antisense oligonucleotides (ASOs). (**C**) Schematic representation of the 5′-leader (nts 1–75) sequence, targeted by a AS-circRNA (1–75) or two antisense oligonucleotides (ASOs, 1–45 and 56–75), either in a luciferase-reporter (luc), or in the viral SARS-CoV-2 context (sgRNAs/genome). Target regions of individual AS-circRNA or ASOs are represented as blue bars with nucleotide coordinates. Secondary structures and regulatory elements are marked (SL1–3, TRS, AUG). Note that the targeting regions of AS_1–75 circRNA and 1–45 ASO omit the first stem-loop (SL1), represented as a dashed line. (**D**) Activity of AS_1–75 circRNA in presence of single point mutations within the 5′-leader: luciferase reporter assays. HeLa cells were transfected with 1–75 AS-circRNA or with control circRNAs. After 24 h, the respective 5′-leader reporter plasmids, either without (WT) or with the indicated point mutations, were transfected, and relative luciferase activities (ratio of Firefly and Renilla expression) were measured, normalized to control circRNAs CTR1 and 2 (mean and standard deviations of three replicates, *P* < 0.001***, two-sided *t*-test). (**E**) Activity of 2′-OMe or 2′-MOE modified ASOs: luciferase reporter assays. HeLa cells were transfected with the AS_1–75 circRNA (1 μg) or ASOs (molar equivalents), respectively. After 24 h, the 5′-leader reporter was transfected, and relative luciferase activities (ratio of Firefly and Renilla expression) were measured, normalized to control circRNAs CTR1 and 2, or correspondingly modified control ASOs CTR1 and 2 (mean and standard deviations of three replicates, *P* < 0.05*, *P* < 0.001***, ns = not significant, two-sided t-test). Fold differences in translational repression between AS_1–75 circRNA and ASO treatments are indicated. (**F**) Antiviral activity of 2′-OMe or 2′-MOE modified ASOs: virus infection assays. Vero E6 cells were transfected with AS_1–75 circRNA (2500 ng per assay) or with ASOs (molar equivalents). After 24 h, cells were infected with SARS-CoV-2 (MOI = 0.1 pfu/cell). The antiviral effects were measured by virus plaque assays at 24 h post-infection (mean and standard deviations of three experiments, *P < 0.05, ns = not significant, two-sided t-test). Untreated (without RNA and transfection reagent) and mock-treated cells (without RNA, but with transfection reagent) served as controls. In addition, control circRNA CTR2 and the correspondingly modified control ASO CTR2 were used. Residual virus titers of significantly affected samples are indicated as ‘percent remaining’ relative to mock treatment, as well as fold differences between circRNA and ASO treatments.

Since newly emerging mutations in the viral genome are of great concern in the current SARS-CoV-2 pandemic, we also assayed whether the activity of designer antisense-circRNA was affected by single point mutations in the target sequence. We focussed on the highly conserved 5′-leader region, where the AS_1–75 circRNA had proved most active, in combination with 5′-leader reporter constructs carrying five different single-point mutations, which naturally occur most frequently in this region (https://www.biosino.org/ViGTK/): 21C→T, 34A**→**T, 35A**→**T, 36C**→**T, 66C**→**T (Figure 5A).

The reduction of reporter activity by AS_1–75 circRNA was not significantly different for the wildtype reporter and for four different mutant versions at positions 21, 34, 35 and 36; only for the 66C→T mutation the antisense activity of AS_1–75 circRNA was reduced, from residual levels of around 25–50% of control levels (Figure 5B–D). These data suggest that the activity of AS_1–75 circRNA is in most cases surprisingly robust and resistant towards single-point mutations.

Finally we compared the activity of AS_1-75 circRNA with corresponding modified, linear antisense oligonucleotides (ASOs), using both the 5′-leader reporter construct as well as viral infection assays (Figure 5BC and EF, respectively). We assayed two different ASOs of standard length, covering positions 1–45 of the 5′-leader sequence (with stem-loop 1 skipped, as in the AS_1–75 circRNA) and positions 56–75, respectively (Figure 5C). These ASOs were modified either by 2′-*O*-methyl (2′-OMe) or 2′-*O*-methoxyethyl (2′-MOE) nucleotides, which should enhance both base-pairing interaction as well as cellular stability. Based on luciferase reporter assays, all four ASOs were clearly less efficient than the AS_1–75 circRNA (by a factor of 3.5–5.4; see Figure 5E). Based on viral infection and virus-titer assays, only the AS_1–75 circRNA, but none of the four ASOs exhibited significant antiviral effects (Figure 5F). We conclude that, comparing antisense-circRNA and state-of-the-art ASOs, these initial assays indicate the superiority of antisense-circRNA as novel and sequence-specific antiviral agents.

## DISCUSSION

To our knowledge, this is the first study to report the design and functional evaluation of a series of AS-circRNAs as a novel tool suitable to interfere with gene expression, applied here to block SARS-CoV-2 proliferation. We focussed on the 5′-UTR region, which is not only highly conserved in sequence and structure, but also absolutely essential for the viral life cycle. Based on a series of AS-circRNAs targeting specific 5′-UTR regions of SARS-CoV-2 genome and sgRNAs, we identified a cap-proximal region (including part of the 5′-leader sequence) as the most effective target region. In particular, AS-circRNAs 1–65 and 1–75 strongly interfered with virus proliferation, resulting in at least 10-fold reduced virus titers. Note that the target sequences of these two antisense-RNAs are discontinuous, omitting the first stem–loop. These data suggest that RNA structure information (if available) should be taken into account in the design and optimization of AS-circRNAs.

When evaluating and comparing the efficiencies of AS-circRNAs in our study, one should also take into account that efficiencies are limited by the circRNA transfection efficiencies under our cell culture conditions; therefore the ‘real’ activities are likely higher than the apparent values determined here. In addition, the experimental conditions of circRNA lipofection, as well as the *in vivo* stabilities of circRNAs can certainly be further improved, for example by systematically optimizing circRNA delivery, evaluating backbone sequences and structure, or introducing RNA modifications or peptide conjugation.

We were able to demonstrate that the inhibitory potency of circular versions of antisense sequences consistently surpassed that of the corresponding linear versions (Figures 2 and 3); moreover, the optimal AS_1–75 circRNA proved superior to state-of-the-art modified ASOs against the same target region, as used in traditional antisense strategies (Figure 5). Most likely this is due to the relatively high metabolic stability of circular RNAs; in addition, structural peculiarities and constraints of how the antisense sequence is exposed in circular configuration may contribute to the activity of AS-circRNAs. Obviously there are more ASO varieties available, as well as combinations of modified nucleotides, that could be tested more systematically, beyond the 2′-OMe and 2′-MOE moieties assayed here. In addition, integrating modified nucleotide positions within synthetic AS-circRNAs appears an attractive and promising option for follow-up studies to further optimize the antisense-circRNA concept.

Our approach to assay the efficiency of antisense-circRNA against SARS-CoV-2 is based on circRNA transfection followed by virus infection, reflecting a prophylactic treatment. However, we have assayed also in the reverse order, viral infection followed by circRNA transfection, and we were able to confirm the efficiency of AS_1–75 circRNA and durability of the antiviral effect (Supplementary Figure S4). This demonstrates that our antisense-circRNA approach is useful not only for prophylactic strategies, but also for protecting against viral infection and for antiviral therapy.

Finally, we were able to confirm the antiviral effect of AS_1–75 circRNA also in an *ex-vivo* cell culture system (Supplementary Figure S5).

What is the mechanistic basis for the strong antiviral effects observed for these AS-circRNAs? Detailed analyses remain to be performed, but will likely reveal a complex picture, because the 5′-terminal region of SARS-CoV-2 is predicted to be involved in multiple levels of the viral replication cycle, such as translational initiation, viral genome replication, synthesis of the 5′-leader-containing subgenomic RNAs, RNA stability, and RNA packaging. Our analysis of viral RNA synthesis (Figure 4) and the data obtained in reporter assays (Figure 2) indicate that the most potent AS-circRNA, AS_1–75, interferes with both the production of subgenomic RNAs and viral protein translation. Effects on other steps are likely but remain to be corroborated by further studies. Initial direct Northern blot assays with reporters argue against the possibility that an RNAi-type cleavage mechanism induced by perfectly base-paired antisense regions is involved. These results, complemented by measuring luciferase mRNA reporter levels via qPCR, rather support a blockage-type mechanism (Supplementary Figure S7).

The extended length of our optimal, unmodified AS_1–75 circRNA confers a certain robustness towards single point mutations in the antisense target region, as we have demonstrated for several naturally occuring point mutations in the leader region (Figure 5). Obviously this represents an added benefit of an AS-circRNA based antiviral strategy, considering the continuously arising mutant SARS-CoV-2 virus forms.

The confirmed functionality of AS-circRNAs designed and characterized in this study in the context of virus infection, including the superior activity of circular over linear RNAs, suggests that circRNAs with antisense functions may exist in nature and play a role in gene regulation. Our results establish designer AS-circRNAs as a new generation of versatile and adjustable RNA therapeutics with significant potential. Finally, in the context of antiviral therapeutic applications, it is worth noting that AS-circRNAs could be easily adjusted to virus escape mutants potentially arising during viral replication and transmission, particularly during virus pandemics of newly emerging viruses.

## DATA AVAILABILITY

RNA-seq data were deposited in the Sequence Read Archive (SRA-ID: PRJNA693241) of NCBI.

## Supplementary Material

gkab1096_Supplemental_File

## References

[1] Perlman S. , Netland J. Coronaviruses post-SARS: update on replication and pathogenesis. Nat. Rev. Microbiol. 2009; 7:439–450. 19430490 10.1038/nrmicro2147 PMC2830095

[2] Perlman S. , Masters P.S. Howley P.M. , Knipe D.M., Whelan S. Coronaviridae: the viruses and their replication. Fields Virology. 2021; I:Philadelphia, PA Wolters Kluwer 410–448.

[3] Madhugiri R. , Karl N., Petersen D., Lamkiewicz K., Fricke M., Wend U., Scheuer R., Marz M., Ziebuhr J. Structural and functional conservation of cis-acting RNA elements in coronavirus 5′-terminal genome regions. Virology. 2018; 517:44–55. 29223446 10.1016/j.virol.2017.11.025 PMC7112051

[4] Miao Z. , Tidu A., Eriani G., Martin F. Secondary structure of the SARS-CoV-2 5′-UTR. RNA Biol. 2021; 18:447–456. 32965173 10.1080/15476286.2020.1814556 PMC7544965

[5] Sola I. , Almazán F., Zúñiga S., Enjuanes L. Continuous and discontinuous RNA synthesis in coronaviruses. Annu. Rev. Virol. 2015; 2:265–288. 26958916 10.1146/annurev-virology-100114-055218 PMC6025776

[6] Kim D. , Lee J.Y., Yang J.S., Kim J.W., Kim V.N., Chang H. The architecture of SARS-CoV-2 transcriptome. Cell. 2020; 181:914–921. 32330414 10.1016/j.cell.2020.04.011 PMC7179501

[7] Zhou P. , Yang X.L., Wang X.G., Hu B., Zhang L., Zhang W., Si H.R., Zhu Y., Li B., Huang C.L. et al. A pneumonia outbreak associated with a new coronavirus of probable bat origin. Nature. 2020; 579:270–273. 32015507 10.1038/s41586-020-2012-7 PMC7095418

[8] Zhu N. , Zhang D., Wang W., Li X., Yang B., Song J., Zhao X., Huang B., Shi W., Lu R. et al. A novel coronavirus from patients with pneumonia in China, 2019. N. Engl. J. Med. 2020; 382:727–733. 31978945 10.1056/NEJMoa2001017 PMC7092803

[9] Polack F.P. , Thomas S.J., Kitchin N., Absalon J., Gurtman A., Lockhart S., Perez J.L., Pérez Marc G., Moreira E.D., Zerbini C et al. Safety and efficacy of the BNT162b2 mRNA Covid-19 vaccine. N. Engl. J. Med. 2020; 383:2603–2615. 33301246 10.1056/NEJMoa2034577 PMC7745181

[10] Bennett C.F. , Swayze E.E. RNA targeting therapeutics: molecular mechanisms of antisense oligonucleotides as a therapeutic platform. Annu. Rev. Pharmacol. Toxicol. 2010; 50:259–293. 20055705 10.1146/annurev.pharmtox.010909.105654

[11] Crooke S.T. , Witztum J.L., Bennett C.F., Baker B.F. RNA-targeted therapeutics. Cell Metab. 2018; 27:714–739. 29617640 10.1016/j.cmet.2018.03.004

[12] Bennett C.F. , Krainer A.R., Cleveland D.W. Antisense oligonucleotide therapies for neurodegenerative diseases. Annu. Rev. Neurosci. 2019; 42:385–406. 31283897 10.1146/annurev-neuro-070918-050501 PMC7427431

[13] Le T.K. , Paris C., Khan K.S., Robson F., Ng W.-L., Rocchi P. Nucleic acid-based technologies targeting coronaviruses. Trends Biochem. Sci. 2021; 46:351–365. 33309323 10.1016/j.tibs.2020.11.010 PMC7691141

[14] Roberts T.C. , Langer R., Wood M.J.A. Advances in oligonucleotide drug delivery. Nat. Rev. Drug Discov. 2020; 19:673–694. 32782413 10.1038/s41573-020-0075-7 PMC7419031

[15] Neuman B.W. , Stein D.A., Kroeker A.D., Churchill M.J., Kim A.M., Kuhn P., Dawson P., Moulton H.M., Bestwick R.K., Iversen P.L. et al. Inhibition, escape, and attenuated growth of severe acute respiratory syndrome coronavirus treated with antisense morpholino oligomers. J. Virol. 2005; 79:9665–9676. 16014928 10.1128/JVI.79.15.9665-9676.2005 PMC1181598

[16] Burrer R. , Neuman B.W., Ting J.P.C., Stein D.A., Moulton H.M., Iversen P.L., Kuhn P., Buchmeier M.J. Antiviral effects of antisense morpholino oligomers in murine coronavirus infection models. J. Virol. 2007; 81:5637–5648. 17344287 10.1128/JVI.02360-06 PMC1900280

[17] Sänger H.L. , Klotz G., Riesner D., Gross H.J., Kleinschmidt A.K. Viroids are single-stranded covalently closed circular RNA molecules existing as highly base-paired rod-likestructures. Proc. Natl. Acad. Sci. U.S.A. 1976; 73:3852–3856. 1069269 10.1073/pnas.73.11.3852 PMC431239

[18] Wilusz J.E. A 360° view of circular RNAs: From biogenesis to functions. Wiley Interdiscip. Rev. RNA. 2018; 9:e1478. 29655315 10.1002/wrna.1478 PMC6002912

[19] Kristensen L.S. , Andersen M.S., Stagsted L.V.W., Ebbesen K.K., Hansen T.B., Kjems J. The biogenesis, biology and characterization of circular RNAs. Nat. Rev. Genet. 2019; 20:675–691. 31395983 10.1038/s41576-019-0158-7

[20] Chen L.L. The expanding regulatory mechanisms and cellular functions of circular RNAs. Nat. Rev. Mol. Cell Biol. 2020; 21:475–490. 32366901 10.1038/s41580-020-0243-y

[21] Salzman J. , Gawad C., Wang P.L., Lacayo N., Brown P.O. Circular RNAs are the predominant transcript isoform from hundreds of human genes in diverse cell types. PLoS One. 2012; 7:e30733. 22319583 10.1371/journal.pone.0030733 PMC3270023

[22] Jeck W.R. , Sorrentino J.A., Wang K., Slevin M.K., Burd C.E., Liu J., Marzluff W.F., Sharpless N.E. Circular RNAs are abundant, conserved, and associated with ALU repeats. RNA. 2013; 19:141–157. 23249747 10.1261/rna.035667.112 PMC3543092

[23] Memczak S. , Jens M., Elefsinioti A., Torti F., Krueger J., Rybak A., Maier L., Mackowiak S.D., Gregersen L.H., Munschauer M. et al. Circular RNAs are a large class of animal RNAs with regulatory potency. Nature. 2013; 495:333–338. 23446348 10.1038/nature11928

[24] Starke S. , Jost I., Rossbach O., Schneider T., Schreiner S., Hung L.-H., Bindereif A. Exon circularization requires canonical splice signals. Cell Rep. 2015; 10:103–111. 25543144 10.1016/j.celrep.2014.12.002

[25] Hansen T.B. , Jensen T.I., Clausen B.H., Bramsen J.B., Finsen B., Damgaard C.K., Kjems J. Natural RNA circles function as efficient microRNA sponges. Nature. 2013; 495:384–388. 23446346 10.1038/nature11993

[26] Piwecka M. , Glažar P., Hernandez-Miranda L.R., Memczak S., Wolf S.A., Rybak-Wolf A., Filipchyk A., Klironomos F., Cerda Jara C.A., Fenske P. et al. Loss of a mammalian circular RNA locus causes miRNA deregulation and affects brain function. Science. 2017; 357:eaam8526. 28798046 10.1126/science.aam8526

[27] Kleaveland B. , Shi C.Y., Stefano J., Bartel D.P. A network of noncoding regulatory RNAs acts in the mammalian brain. Cell. 2018; 174:350–362. 29887379 10.1016/j.cell.2018.05.022 PMC6559361

[28] Hentze M.W. , Preiss T. Circular RNAs: splicing's enigma variations. EMBO J. 2013; 32:923–925. 23463100 10.1038/emboj.2013.53 PMC3616293

[29] Jost I. , Shalamova L.A., Gerresheim G.K., Niepmann M., Bindereif A., Rossbach O. Functional sequestration of microRNA-122 from Hepatitis C Virus by circular RNA sponges. RNA Biol. 2018; 15:1032–1039. 29486652 10.1080/15476286.2018.1435248 PMC6161685

[30] Müller S. , Wedler A., Breuer J., Glaß M., Bley N., Lederer M., Haase J., Misiak C., Fuchs T., Ottmann A. et al. Synthetic circular miR-21 RNA decoys enhance tumor suppressor expression and impair tumor growth in mice. NAR Cancer. 2020; 2:zcaa014. 34316687 10.1093/narcan/zcaa014 PMC8210135

[31] Schreiner S. , Didio A., Hung L.-H., Bindereif A. Design and application of circular RNAs with protein-sponge function. Nucleic Acids Res. 2020; 48:12326–12335. 33231682 10.1093/nar/gkaa1085 PMC7708053

[32] Litke J.L. , Jaffrey S.R. Highly efficient expression of circular RNA aptamers in cells using autocatalytic transcripts. Nat. Biotechnol. 2019; 37:667–675. 30962542 10.1038/s41587-019-0090-6 PMC6554452

[33] Breuer J. , Rossbach O. Production and purification of artificial circular RNA sponges for application in molecular biology and medicine. Methods Protoc. 2020; 3:42. 10.3390/mps3020042 PMC7359697 32466614

[34] Medenbach J. , Seiler M., Hentze M.W. Translational control via protein-regulated upstream open reading frames. Cell. 2011; 145:902–913. 21663794 10.1016/j.cell.2011.05.005

[35] Müller C. , Schulte F.W., Lange-Grünweller K., Obermann W., Madhugiri R., Pleschka S., Ziebuhr J., Hartmann R.K., Grünweller A. Broad-spectrum antiviral activity of the eIF4A inhibitor silvestrol against corona- and picornaviruses. Antivir. Res. 2018; 150:123–129. 29258862 10.1016/j.antiviral.2017.12.010 PMC7113723

[36] Schneider T. , Schreiner S., Preußer C., Bindereif A., Rossbach O. Northern blot analysis of circular RNAs. Methods Mol. Biol. 2018; 1724:119–133. 29322445 10.1007/978-1-4939-7562-4_10

[37] Dobin A. , Davis C.A., Schlesinger F., Drenkow J., Zaleski C., Jha S., Batut P., Chaisson M., Gingeras T.R. STAR: ultrafast universal RNA-seq aligner. Bioinformatics. 2013; 29:15–21. 23104886 10.1093/bioinformatics/bts635 PMC3530905

[38] Ziv O. , Price J., Shalamova L., Kamenova T., Goodfellow I., Weber F., Miska E.A. The Short- and Long-Range RNA-RNA Interactome of SARS-CoV-2. Mol. Cell. 2020; 80:1067–1077. 33259809 10.1016/j.molcel.2020.11.004 PMC7643667

[39] Kim D. , Kim S., Park J., Chang H.R., Chang J., Ahn J., Park H., Park J., Son N., Kang G. et al. A high-resolution temporal atlas of the SARS-CoV-2 translatome and transcriptome. Nat. Commun. 2021; 12:5120. 34433827 10.1038/s41467-021-25361-5 PMC8387416

[40] Cao C. , Cai Z., Xiao X., Rao J., Chen J., Hu N., Yang M., Xing X., Wang Y., Li M. et al. The architecture of the SARS-CoV-2 RNA genome inside virion. Nat. Commun. 2021; 12:3917. 34168138 10.1038/s41467-021-22785-x PMC8225788

[41] Boulant S. , Stanifer M., Lozach P.Y. Dynamics of virus-receptor interactions in virus binding, signaling, and endocytosis. Viruses. 2015; 7:2794–2815. 26043381 10.3390/v7062747 PMC4488714

[42] Jonsdottir H.R. , Dijkman R. Coronaviruses and the human airway: a universal system for virus-host interaction studies. Virol. J. 2016; 13:24. 26852031 10.1186/s12985-016-0479-5 PMC4744394

