# Supplementary material for: Inhibition of SARS-CoV-2 coronavirus proliferation by designer antisense-circRNAs
Source: Nucleic Acids Res. 2021 Nov 24;49(21):12502–16. doi: 10.1093/nar/gkab1096 (PMC8643703; doi:10.1093/nar/gkab1096)
Supplement: gkab1096_Supplemental_File [file gkab1096_supplemental_file.pdf]

Supplementary Material

Inhibition of SARS-CoV-2 coronavirus proliferation by designer antisense-circRNAs

Christina Pfafenrot [1,3], Tim Schneider [1,3], Christin Müller [2,3], Lee-Hsueh Hung [1], Silke Schreiner [1], John Ziebuhr [2], and Albrecht Bindereif [1]*

[1] Institute of Biochemistry, Justus Liebig University of Giessen, 35392 Giessen, Germany
[2] Institute of Medical Virology, Justus Liebig University of Giessen, 35392 Giessen, Germany

* corresponding author, e-mail: albrecht.bindereif@chemie.bio.uni-giessen.de
[3] These authors contributed equally.

Supplementary Material comprises seven Supplementary Figures and one Supplementary Table.

Supplementary Figure S1.

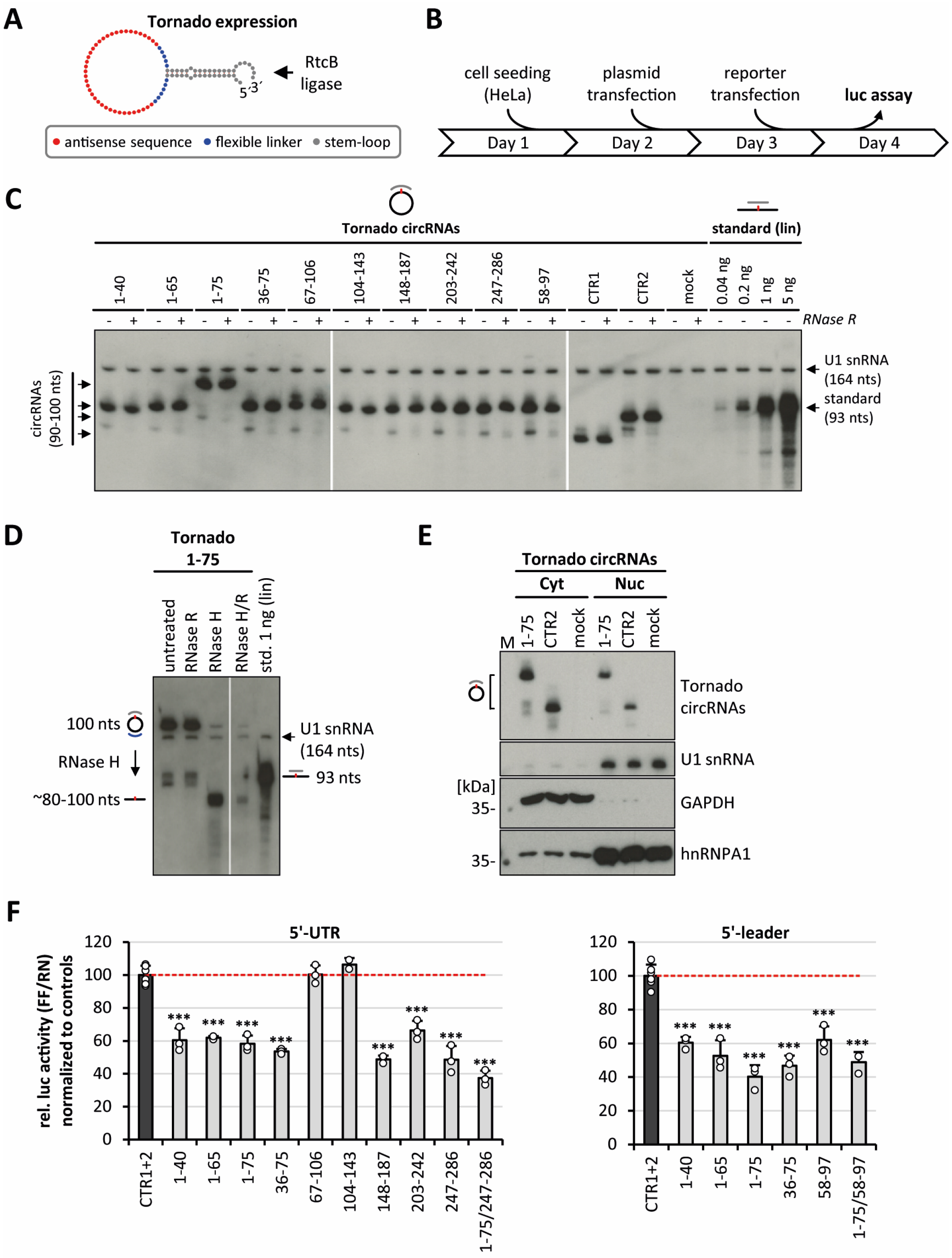

Supplementary Figure S1.

Tornado-based expression of AS-circRNAs in HeLa cells and screening of antisense activities by 5’-UTR and 5’-leader reporter assays.

(A) Design of AS-circRNAs expressed in mammalian cells by the so-called Tornado system (Litke and Jaffrey, 2019), based on RNA-polymerase III-driven expression, ribozyme-mediated processing, and *in vivo* circularization by endogenous RtcB tRNA ligase. Each circular RNA is composed of a stem-loop (grey), a short flexible linker (blue), and the antisense sequence (red).

(B) Experimental workflow for luciferase reporter assays in HeLa cells overexpressing AS-circRNAs.

(C) Tornado-based overexpression of AS-circRNAs in HeLa cells (as indicated) and detection by Northern blot analysis, using a circular-junction specific common probe. Circularity was confirmed by RNase R treatment (-/+). As an input control, U1 snRNA was detected by a U1-specific Northern probe. In addition, a linear standard RNA containing the circular junction (0.04 to 5 ng) served to quantitate Northern signals.

(D) Additional evidence for circularity of Tornado-expressed AS-circRNAs. Total RNA from HeLa cells overexpressing AS_1-75 circRNA was analyzed by Northern blot, using a circular-junction specific probe: RNA aliquots were either left untreated, RNase R digested, treated with RNase H and a junction-specific DNA oligonucleotide (resulting in a cleavage product of ~79 nucleotides), or combining RNase R and H treatment. In addition, 1 ng of the linear standard RNA was applied.

(E) Cellular distribution of Tornado-expressed AS-circRNAs. HeLa cells overexpressing either AS_1-75 or CTR2 control circRNAs were fractionated into cytoplasmic and nuclear fractions, followed by RNA preparation and Northern blot analysis with a common circular-junction probe. In addition, RNA from mock-treated cells were analyzed. For control and as fractionation markers, nuclear U1 snRNA and hnRNP A1 protein, as well as cytoplasmic GAPDH protein were detected by Northern and Western blot.

(F) Translational repression of SARS-CoV-2 5´-UTR and 5´-leader reporter constructs by AS-circRNAs. HeLa cells were transfected with the respective circRNA (as indicated below the diagram) or a combination thereof (e.g. AS_1-40/247-286). After 24 h, the respective reporter was transfected (5’-UTR or 5’-leader), and relative luciferase activities (ratio of Firefly and Renilla expression) were measured, normalized to control circRNAs CTR1 and 2 (mean and standard deviations of three replicates, $p < 0.05$*, $p < 0.005$**, $p < 0.001$***, ns = not significant, two-sided t-test). Source data are provided as a Source Data file.

Supplementary Figure S2.

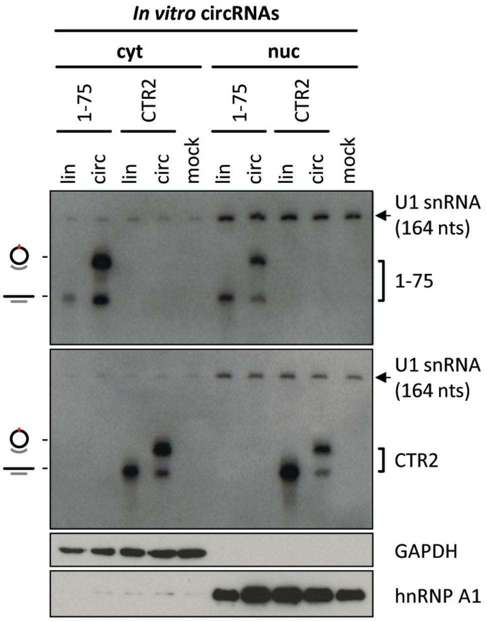

Supplementary Figure S2.
Northern analysis of RNAs transfected in HeLa cells.
Northern blot analysis of fractionated HeLa cells transfected with synthetic AS_1-75 and CTR2 control RNAs, each in linear or circular configuration, or after mock treatment. 24 h after circRNA transfection, cells were fractionated and RNA was prepared, followed by Northern blot analysis (in equivalent quantities), using AS_1-75 (top) and CTR2-specific probes (bottom), which detect both linear and circular forms. As input control, and for confirming nuclear-cytoplasmic fractionation, U1 snRNA was detected by Northern blot, as well as the nuclear hnRNP A1 and the cytoplasmic GAPDH protein by Western blot. Source data are provided as a Source Data file.

Supplementary Figure S3.

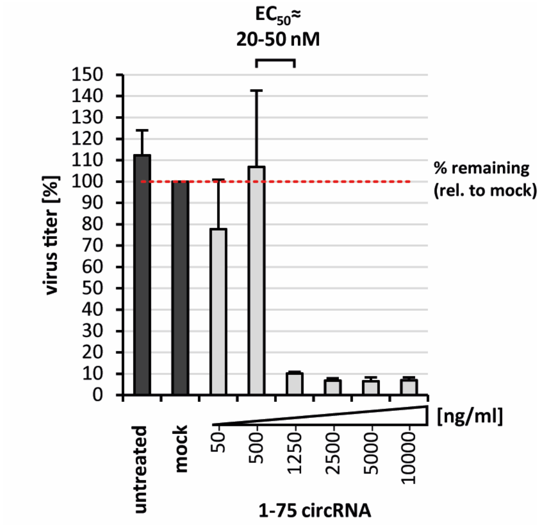

Supplementary Figure S3.
EC_{50} estimation for AS_1-75 circRNA.
EC_{50} value for AS_1-75 circRNA in virus infection assays. Based on combining our data from virus titer assays in Vero E6 cells transfected with different quantities of AS_1-75 circRNA (see **Figures 3BD, Figure 5F and Supplementary Figure S6**), an EC_{50} value between 500 and 1250 ng/ml (20-50 nM) was estimated (mean and SEM of combined data). Source data are provided as a Source Data file.

Supplementary Figure S4.

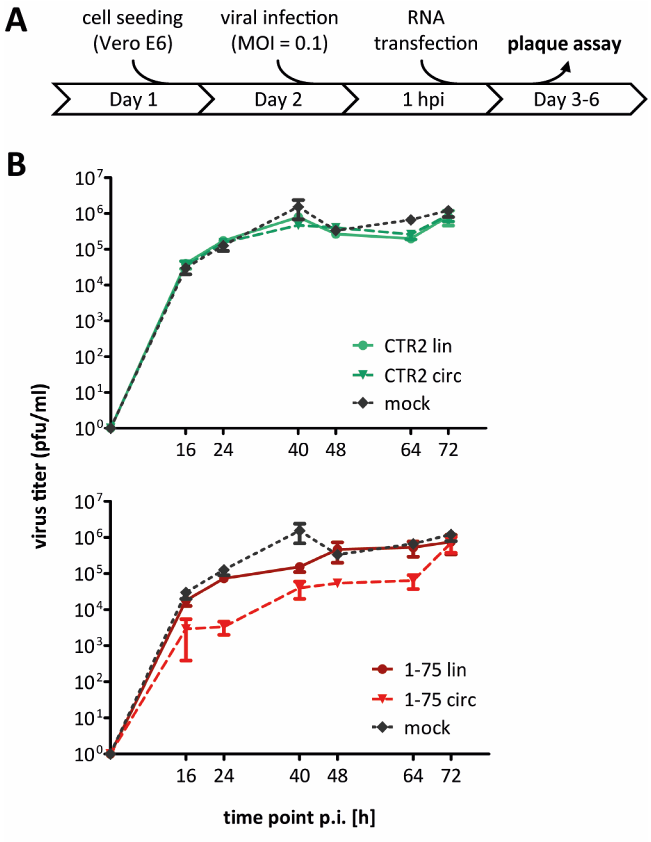

Supplementary Figure S4.
Inhibition of SARS-CoV-2 proliferation by AS-circRNAs transfected post-infection.
(A) Experimental workflow for viral infection assays using Vero E6 cells, transfected with synthetic RNAs (circRNA versus linear RNA) after infection with SARS-CoV-2.
(B) Durability of antiviral activity of AS_1-75 circRNA. Vero E6 cells were infected with SARS-CoV-2 (MOI = 0.1 pfu/cell), followed one hour post-infection by transfection of AS_1-75 circRNA or its linear counterpart (bottom panel; in red; mean and SEM of three experiments). Plaque assays were performed to determine virus titers in culture supernatants collected at the indicated time points (16 to 72 h post-transfection). As controls, mock-treated cells (without RNA, but with transfection reagent) were used (top and bottom panels; in black), as well as transfections with linear or circular control RNA (CTR2; top panel; in green).

Supplementary Figure S5.

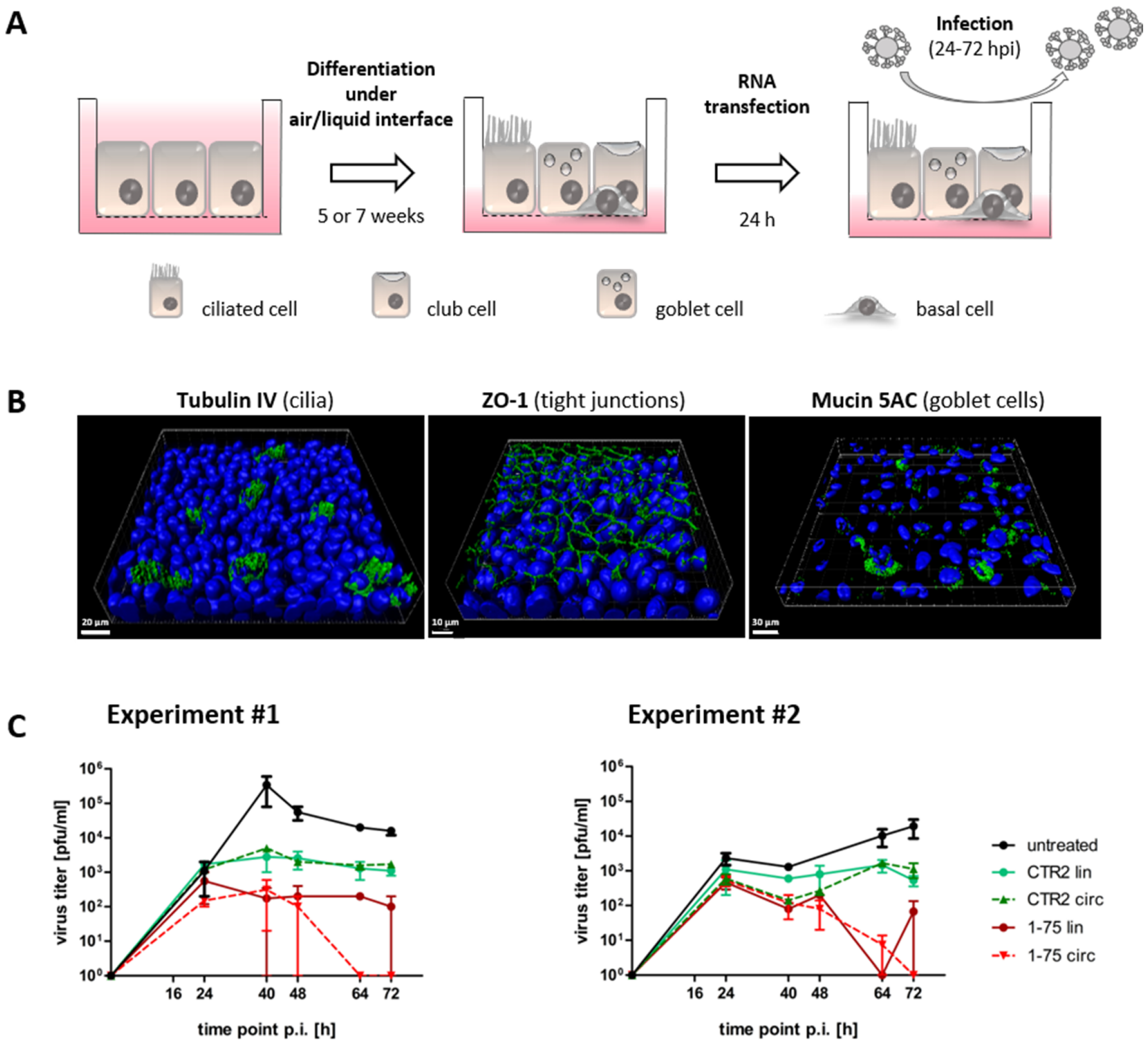

Supplementary Figure S5.
Inhibition of SARS-CoV-2 proliferation by AS-circRNA in primary normal human bronchial epithelial (NHBE) cells
(A) Experimental workflow for viral infection assays using NHBE cells. After reaching confluence, the cells were cultivated under air-liquid conditions for five or seven weeks to allow differentiation into pseudo-stratified human airway epithelia, followed by RNA transfection, virus infection, and plaque assays.
(B) Representative 3D models for validation of the differentiation status of NHBE cells via immunofluorescence analysis, using antibodies against tubulin IV, ZO-1 and mucin 5AC.
(C) Durability of antiviral activity of AS-1-75 circRNA. Differentiated NHBE cells (five weeks: experiment #1; seven weeks: experiment #2) were transfected with AS_1-75 circRNA or its linear counterpart (in red), followed by viral infection (MOI = 3 pfu/cell) after 24 h (mean and SEM of two, Experiment #1, or three, Experiment #2, technical replicates). Plaques assays were performed to determine virus titers in culture supernatants collected at the indicated time points (24-72 h post-infection). As controls, untreated cells (in black) were used, as well as transfections with linear and circular control RNA (CTR2; in green).

Supplementary Figure S6.

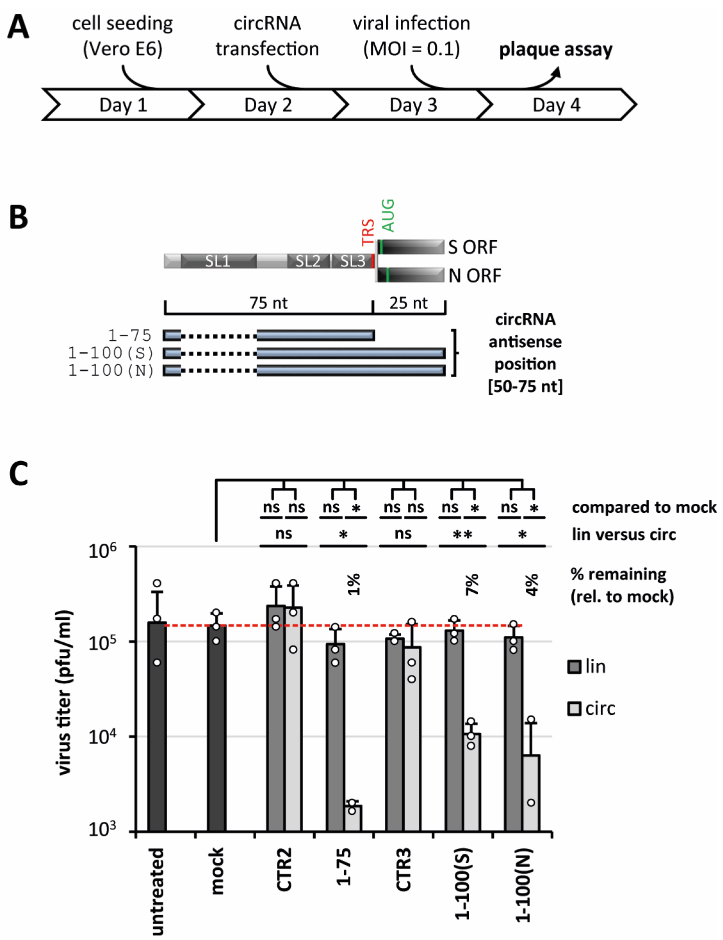

Supplementary Figure S6.
Inhibition of SARS-CoV-2 proliferation by elongated AS-circRNAs: viral infection assays with AS_1-100 (S) and AS_1-100 (N) circRNAs.
(A) Experimental workflow for viral infection assays using Vero E6 cells.
(B) Schematic representation of the S- and N-protein-encoding subgenomic RNAs, including the 75-nucleotide 5'-leader with its stem-loop elements (SL1-3), the transcription regulatory sequence (TRS), and the S- or N-ORF. The two elongated AS-circRNAs are represented below, targeting specifically the S or N subgenomic RNAs, and consisting of 75 nts against the 5'-leader sequence (omitting SL1; dashed line) and 25 nts of 5'-terminal S- or N-ORF. In addition, AS_1-75 RNA (lin/circ) is represented, which was used for comparison.
(C) Viral infection assays with elongated AS-circRNAs. Vero E6 cells were transfected with AS_1-100 (S) and AS_1-100 (N) circRNAs, both in linear or circular form (2500 ng per assay), followed by SARS-CoV-2 viral infection (MOI = 0.1 pfu/cell) and, 24 h post-infection, by virus titer assays (mean and standard deviations of three experiments, $p < 0.05$*, ns = not significant, two-sided t-test). As controls and for comparison, untreated (without RNA and transfection reagent) and mock-treated cells (without RNA, but with transfection reagent), as well as cells transfected with CTR2 and CTR3 control, or with AS_1-75 linear and circular RNAs, were included. Residual virus titers of significantly affected samples are indicated as "percent remaining" relative to mock treatment. Source data are provided as a Source Data file.

Supplementary Figure S7.

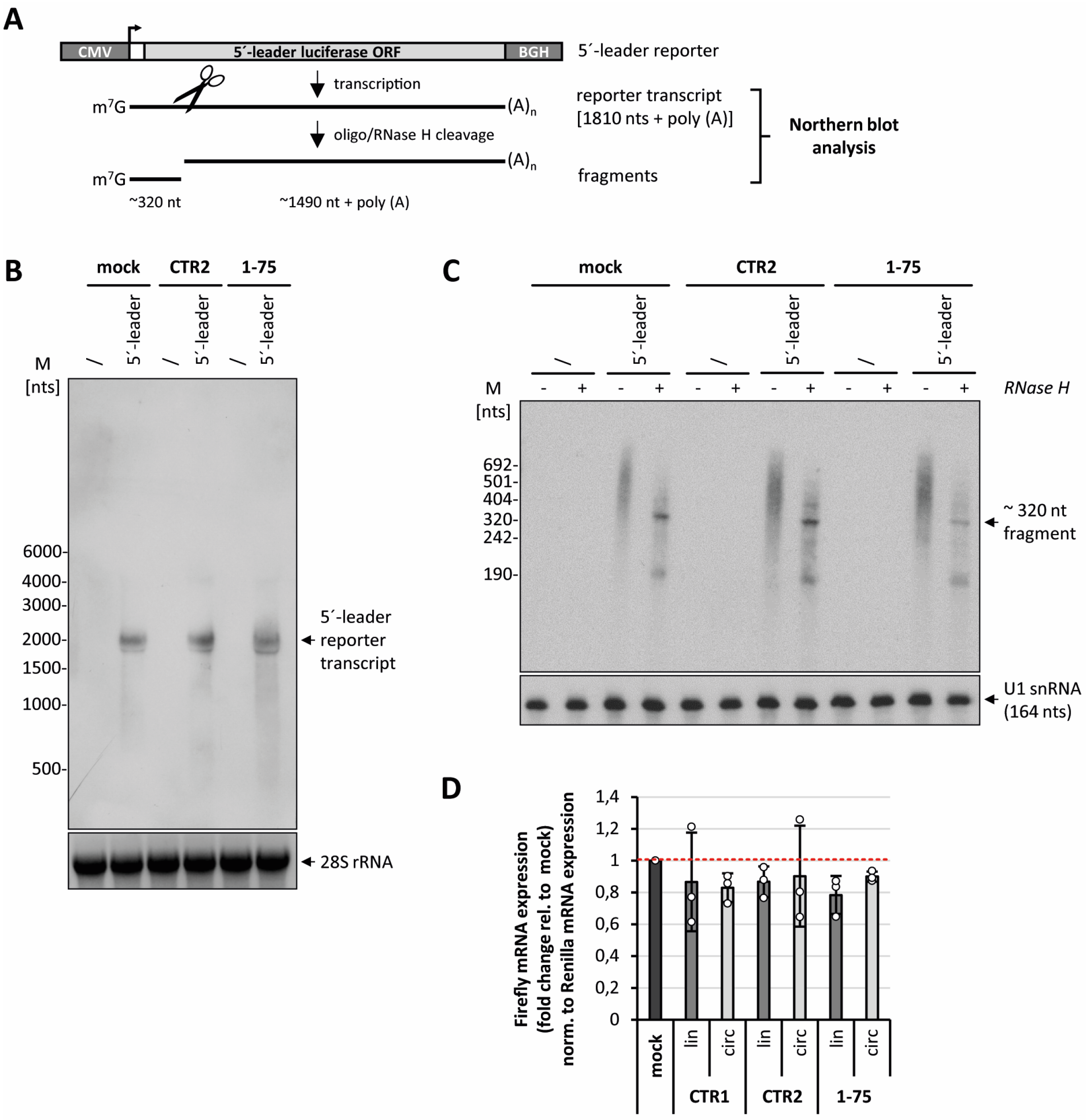

Supplementary Figure S7.
Steric blocking versus cleavage: evidence against an antisense-based cleavage mechanism of AS_1-75 circRNA.
(A) Experimental strategy. The 5´-leader reporter construct is expressed as a capped and polyadenylated mRNA that can be cleaved into two mRNA fragments of characteristic sizes, based on RNase H and a complementary DNA oligonucleotide. Subsequent Northern blot reveals whether the transcript is stable upon AS-1-75 circRNA transfection (evidence for steric blocking) or degraded (evidence for cleavage mechanism).
(B) Northern blot analysis of full-length 5´-leader reporter transcript. HeLa cells were transfected with either no RNA (mock), with 5 µg control (CTR2), or with AS_1-75 circRNA. On the next day, cells were mock-transfected (/ = no plasmid), or transfected with the 5´-leader reporter construct. At 24 h post-transfection, total RNA was prepared and subjected to glyoxal-Northern blot analysis, to detect the 5´-leader reporter transcript [1,810 nts + poly(A)]. *M*, RiboRuler High Range RNA Ladder (Thermo Fisher Scientific). As input control, 28S rRNA was detected by ethidium bromide staining.
(C) Northern blot analysis of RNase H-cleaved fragment of 5´-leader reporter transcript. Total RNA (3 µg each) obtained after mock, control circRNA, or AS_1-75 circRNA transfections (as described in panel **B**) was additionally subjected to RNase H cleavage, followed by Northern blot analysis (denaturing PAGE). The expected cleavage fragment of ~320 nts (see panel **A**) was detected in all RNase H (+) reactions, independently of the circRNA transfected (CTR2 or AS_1-75), and also in the mock transfection. *M*, DNA molecular weight marker, DIG-labelled (Roche). As input control, U1 snRNA was detected by Northern blot. Source data are provided as a Source Data file.
(D) Detection of Firefly mRNA levels by RT-qPCR. HeLa cells were transfected with 1 µg linear or circular RNA. After 24 h, the respective reporter plasmids were transfected (Firefly and Renilla), and relative expression levels of the reporter mRNAs (Firefly mRNA expression normalized to Renilla mRNA expression) were determined by RT-qPCR in comparison to mock transfection (only reporter plasmid transfection, without RNA transfection). Mean and standard deviations are based on three experiments.

Supplementary Table S1.

Tornado circRNAs

Name		Oligonucleotides (with NotI and SacII overhangs) 5´-3´	Final circRNA sequence 5´-3´
1-40	_fwd<br>_rev	GGCCGCAAGTTGGTTGGTTAGTTACCTGGGAAGGTATAAACCTTTAATAAAAACCGC<br>GGTTTTTATTAAAGGTTTATACCTTCCCAGGTAACTAACCAACCAACTTGC	AACCATGCCGAGTGCGGCCGCAAGTTGGTTGGTTAGTTACCTGGGAAGGTATAAACCTTTAATAAAAACCGCGGTCGGCGTGGACTGTAG
1-65	_fwd<br>_rev	GGCCGCAAAGAACAGATCTACAAGAGATCGAAAGTTGGTTaaTTTAATAAAAACCGC<br>GGTTTTTATTAAAttAACCAACTTTCGATCTCTTGTAGATCTGTTCTTTGC	AACCATGCCGAGTGCGGCCGCAAAGAACAGATCTACAAGAGATCGAAAGTTGGTTaaTTTAATAAAAACCGCGGTCGGCGTGGACTGTAG
1-75	_fwd<br>_rev	GGCCGCAAGTTCGTTTAGAGAACAGATCTACAAGAGATCGAAAGTTGGTTaaTTTAATAAAAACCGC<br>GGTTTTTATTAAAttAACCAACTTTCGATCTCTTGTAGATCTGTTCTCTAAACGAACTTGC	AACCATGCCGAGTGCGGCCGCAAGTTCGTTTAGAGAACAGATCTACAAGAGATCGAAAGTTGGTTaaTTTAATAAAAACCGCGGTCGGCGTGGACTGTAG
36-75	_fwd<br>_rev	GGCCGCAAGTTCGTTTAGAGAACAGATCTACAAGAGATCGAAAGTTGGAAAAACCGC<br>GGTTTTTCCAACTTTCGATCTCTTGTAGATCTGTTCTCTAAACGAACTTGC	AACCATGCCGAGTGCGGCCGCAAGTTCGTTTAGAGAACAGATCTACAAGAGATCGAAAGTTGGAAAAACCGCGGTCGGCGTGGACTGTAG
58-97	_fwd<br>_rev	GGCCGCAATAAAACAAGAAAAACAAACATAGTTCGTTTAGAGAACAGAAAAAACCGC<br>GGTTTTTTCTGTTCTCTAAACGAACTATGTTTGTTTTTCTTGTTTTATTGC	AACCATGCCGAGTGCGGCCGCAATAAAACAAGAAAAACAAACATAGTTCGTTTAGAGAACAGAAAAAACCGCGGTCGGCGTGGACTGTAG
67-106	_fwd<br>_rev	GGCCGCAAGCAGCCGAGTGACAGCCACACAGATATTAAAGTTCGTTTAAAAAACCGC<br>GGTTTTTTAAACGAACTTTAATATCTGTGTGGCTGTCACTCGGCTGCTTGC	AACCATGCCGAGTGCGGCCGCAAGCAGCCGAGTGACAGCCACACAGATATTAAAGTTCGTTTAAAAAACCGCGGTCGGCGTGGACTGTAG
104-143	_fwd<br>_rev	GGCCGCAATTAGATATTAATTATACTGCGTGAGTGCACTAAGCATGCAAAAAACCGC<br>GGTTTTTTGCATGCTTAGTGCACTCACGCAGTATAATTAATATCTAATTGC	AACCATGCCGAGTGCGGCCGCAATTAGATATTAATTATACTGCGTGAGTGCACTAAGCATGCAAAAAACCGCGGTCGGCGTGGACTGTAG
148-187	_fwd<br>_rev	GGCCGCAATGCAGAAGATAGACGAGTTACTCGTGTCCTGTCAACGACAAAAAACCGC<br>GGTTTTTTGTCGTTGACAGGACACGAGTAACTCGTCTATCTTCTGCATTGC	AACCATGCCGAGTGCGGCCGCAATGCAGAAGATAGACGAGTTACTCGTGTCCTGTCAACGACAAAAAACCGCGGTCGGCGTGGACTGTAG
203-242	_fwd<br>_rev	GGCCGCAACGAAACCTAGATGTGCTGATGATCGGCTGCAACACGGACGAAAAACCGC<br>GGTTTTTCGTCCGTGTTGCAGCCGATCATCAGCACATCTAGGTTTCGTTGC	AACCATGCCGAGTGCGGCCGCAACGAAACCTAGATGTGCTGATGATCGGCTGCAACACGGACGAAAAACCGCGGTCGGCGTGGACTGTAG
247-286	_fwd<br>_rev	GGCCGCAAACCAGGGACAAGGCTCTCCATCTTACCTTTCGGTCACACCAAAAACCGC<br>GGTTTTTGGTGTGACCGAAAGGTAAGATGGAGAGCCTTGTCCCTGGTTTGC	AACCATGCCGAGTGCGGCCGCAAACCAGGGACAAGGCTCTCCATCTTACCTTTCGGTCACACCAAAAACCGCGGTCGGCGTGGACTGTAG
CTR1	_fwd<br>_rev	GGCCGCAAAGATGCGCACCGCACAGATGCGCACCACGCCACGCGTAGAAAAAAACCGC<br>GGTTTTTTCTACGCGTGGCGTGGTGCGCATCTGTGCGGTGCGCATCTTTGC	AACCATGCCGAGTGCGGCCGCAAAGATGCGCACCGCACAGATGCGCACCACGCCACGCGTAGAAAAAACCGCGGTCGGCGTGGACTGTAG
CTR2	_fwd<br>_rev	GGCCGCAAAGTCAGTGAGTCAGTGTAATACGGGAGGATACCCGCTGTCAAAAACCGC<br>GGTTTTTGACAGCGGGTATCCTCCCGTATTACACTGACTCACTGACTTTGC	AACCATGCCGAGTGCGGCCGCAAAGTCAGTGAGTCAGTGTAATACGGGAGGATACCCGCTGTCAAAAACCGCGGTCGGCGTGGACTGTAG
			**Legend**<br>AACCATGCCGAGTGCGGCCGCAA**NNNNNNNNNNNNNNNNNNNNNNNNNNNNNNNNnnNNNNNN**AAAAACCGCGGTCGGCGTGGACTGTAG<br>Tornado stem loop, flexible linker, **N** = antisense sequence, **N** = T->A (for extended T-stretches), **nn** = base of SL1

***In vitro* produced RNA**

Name		Oligonucleotides 5´-3´	Final circRNA sequence 5´-3´
1-40	_fwd<br>_rev	TAATACGACTCACTATAGGGAGTAAGCAAAGTTGGTTGGTTTGTTACCTGGGAAGGTATAAACCTTTAATAAAGCTTACAGTA<br>TACTGTAAGCTTTATTAAAGGTTTATACCTTCCCAGGTAACAAACCAACCAACTTTGCTTACTCCCTATAGTGAGTCGTATTA	GGGAGTAAGCAAAGTTGGTTGGTTTGTTACCTGGGAAGGTATAAACCTTTAATAAAGCTTACAGTA
1-65	_fwd<br>_rev	TAATACGACTCACTATAGGGAGTAAGCAAAAGAACAGATCTACAAGAGATCGAAAGTTGGTTaaTTTAATAAAGCTTACAGTA<br>TACTGTAAGCTTTATTAAAttAACCAACTTTCGATCTCTTGTAGATCTGTTCTTTTGCTTACTCCCTATAGTGAGTCGTATTA	GGGAGTAAGCAAAAGAACAGATCTACAAGAGATCGAAAGTTGGTTaaTTTAATAAAGCTTACAGTA
1-75	_fwd<br>_rev	TAATACGACTCACTATAGGGAGTAAGCAAAGTTCGTTTAGAGAACAGATCTACAAGAGATCGAAAGTTGGTTaaTTTAATAAAGCTTACAGTA<br>TACTGTAAGCTTTATTAAAttAACCAACTTTCGATCTCTTGTAGATCTGTTCTCTAAACGAACTTTGCTTACTCCCTATAGTGAGTCGTATTA	GGGAGTAAGCAAAGTTCGTTTAGAGAACAGATCTACAAGAGATCGAAAGTTGGTTaaTTTAATAAAGCTTACAGTA
1-100(S)	_fwd<br>_rev	TAATACGACTCACTATAGGGAGTAAGCAAACAATAAAACAAGAAAAACAAACATTGTTCGTTTAGAGAACAGATCTACAAGAGATCGAAAGTTGGTTaaTTTAATAAAGCTTACAGTA<br>TACTGTAAGCTTTATTAAAttAACCAACTTTCGATCTCTTGTAGATCTGTTCTCTAAACGAACAATGTTTGTTTTTCTTGTTTTATTGTTTGCTTACTCCCTATAGTGAGTCGTATTA	GGGAGTAAGCAAACAATAAAACAAGAAAAACAAACATTGTTCGTTTAGAGAACAGATCTACAAGAGATCGAAAGTTGGTTaaTTTAATAAAGCTTACAGTA
1-100(N)	_fwd<br>_rev	TAATACGACTCACTATAGGGAGTAAGCAAAGGTCCATTATCAGACATTTTAGTTTGTTCGTTTAGAGAACAGATCTACAAGAGATCGAAAGTTGGTTaaTTTAATAAAGCTTACAGTA<br>TACTGTAAGCTTTATTAAAttAACCAACTTTCGATCTCTTGTAGATCTGTTCTCTAAACGAACAAACTAAAATGTCTGATAATGGACCTTTGCTTACTCCCTATAGTGAGTCGTATTA	GGGAGTAAGCAAAGGTCCATTATCAGACATTTTAGTTTGTTCGTTTAGAGAACAGATCTACAAGAGATCGAAAGTTGGTTaaTTTAATAAAGCTTACAGTA
36-75	_fwd<br>_rev	TAATACGACTCACTATAGGGAGTAAGCAAAGTTCGTTTAGAGAACAGATCTACAAGAGATCGAAAGTTGGAAAGCTTACAGTA<br>TACTGTAAGCTTTCCAACTTTCGATCTCTTGTAGATCTGTTCTCTAAACGAACTTTGCTTACTCCCTATAGTGAGTCGTATTA	GGGAGTAAGCAAAGTTCGTTTAGAGAACAGATCTACAAGAGATCGAAAGTTGGAAAGCTTACAGTA
58-97	_fwd<br>_rev	TAATACGACTCACTATAGGGAGTAAGCAAATAAAACAAGAAAAACAAACATTGTTCGTTTAGAGAACAGAAAAGCTTACAGTA<br>TACTGTAAGCTTTTCTGTTCTCTAAACGAACAATGTTTGTTTTTCTTGTTTTATTTGCTTACTCCCTATAGTGAGTCGTATTA	GGGAGTAAGCAAATAAAACAAGAAAAACAAACATTGTTCGTTTAGAGAACAGAAAAGCTTACAGTA
247-286	_fwd<br>_rev	TAATACGACTCACTATAGGGAGTAAGCAAAACCAGGGACAAGGCTCTCCATCTTACCTTTCGGTCACACCAAAGCTTACAGTA<br>TACTGTAAGCTTTGGTGTGACCGAAAGGTAAGATGGAGAGCCTTGTCCCTGGTTTGCTTACTCCCTATAGTGAGTCGTATTA	GGGAGTAAGCAAAACCAGGGACAAGGCTCTCCATCTTACCTTTCGGTCACACCAAAGCTTACAGTA
CTR1	_fwd<br>_rev	TAATACGACTCACTATAGGGAGTAAGCAAAAGATGCGCACCGCACAGATGCGCACCACGCCACGCGTAGAAAAGCTTACAGTA<br>TACTGTAAGCTTTTCTACGCGTGGCGTGGTGCGCATCTGTGCGGTGCGCATCTTTTGCTTACTCCCTATAGTGAGTCGTATTA	GGGAGTAAGCAAAAGATGCGCACCGCACAGATGCGCACCACGCCACGCGTAGAAAAGCTTACAGTA
CTR2	_fwd<br>_rev	TAATACGACTCACTATAGGGAGTAAGCAAAAGTCAGTGAGTCAGTGTAATACGGGAGGATACCCGCTGTCAAAGCTTACAGTA<br>TACTGTAAGCTTTGACAGCGGGTATCCTCCCGTATTACACTGACTCACTGACTTTGCTTACTCCCTATAGTGAGTCGTATTA	GGGAGTAAGCAAAAGTCAGTGAGTCAGTGTAATACGGGAGGATACCCGCTGTCAAAGCTTACAGTA
CTR3	_fwd<br>_rev	TAATACGACTCACTATAGGGAGTAAGCAAACTGTCGCCCATAGGAGGGCATAATGTGACTGAGTGACTGACTGTCGCCCATAGGAGGGCATAATGTGACTGAGTGAAAGCTTACAGTA<br>TACTGTAAGCTTTCACTCAGTCACATTATGCCCTCCTATGGGCGACAGTCAGTCACTCAGTCACATTATGCCCTCCTATGGGCGACAGTTTGCTTACTCCCTATAGTGAGTCGTATTA	GGGAGTAAGCAAACTGTCGCCCATAGGAGGGCATAATGTGACTGAGTGACTGACTGTCGCCCATAGGAGGGCATAATGTGACTGAGTGAAAGCTTACAGTA
			**Legend**<br>GGGAGTAAGCAAA**NNNNNNNNNNNNNNNNNNNNNNNNNNNNNNNNnnNNNNNN**AAAGCTTACAGTA<br>overhang, stem loop, flexible linker, **N** = antisense sequence, **nn** = base of SL1

Northern Blot probes

Name		Oligonucleotides 5´-3´	Riboprobe sequence 5´-3´
Tornado junction	_fwd<br>_rev	TAATACGACTCACTATAGGGTTGCGGCCGCACTCGGCATGGTTCTACAGTCCACGCCGACCGCGGTTTTTTTTTTTTTTTTTTT<br>AAAAAAAAAAAAAAAAAAAACCGCGGTCGGCGTGGACTGTAGAACCATGCCGAGTGCGGCCGCAACCCTATAGTGAGTCGTATTA	GGGTTGCGGCCGCACTCGGCATGGTTCTACAGTCCACGCCGACCGCGGTTTTTTTTTTTTTTTTTTT
1-75	_fwd<br>_rev	TAATACGACTCACTATAGGGTAACCAACTTTCGATCTCTTGTAGATCTGTTCTCTAAACGAACTTTTTTTTTTTTTTTTTTTTTTT<br>AAAAAAAAAAAAAAAAAAAAAAAAGTTCGTTTAGAGAACAGATCTACAAGAGATCGAAAGTTGGTTACCCTATAGTGAGTCGTATTA	GGGTAACCAACTTTCGATCTCTTGTAGATCTGTTCTCTAAACGAACTTTTTTTTTTTTTTTTTTTTTTT
CTR2	_fwd<br>_rev	TAATACGACTCACTATAGGGTTTGACAGCGGGTATCCTCCCGTATTACACTGACTCACTGACTTTTTTTTTTTTTTTTTTTTTTT<br>AAAAAAAAAAAAAAAAAAAAAAAAAGTCAGTGAGTCAGTGTAATACGGGAGGATACCCGCTGTCAAACCCTATAGTGAGTCGTATTA	GGGTTTGACAGCGGGTATCCTCCCGTATTACACTGACTCACTGACTTTTTTTTTTTTTTTTTTTTTTT
U1 snRNA	_fwd<br>_rev	TAATACGACTCACTATAGGGATACTTACCTGGCAGGGGAGATACCATGATTTTTTTTTTTTTTTTTTTT<br>AAAAAAAAAAAAAAAAAAAAATCATGGTATCTCCCCTGCCAGGTAAGTATCCCTATAGTGAGTCGTATTA	GGGATACTTACCTGGCAGGGGAGATACCATGATTTTTTTTTTTTTTTTTTTT
SARS-CoV-2 genome/sgRNAs	_fwd<br>_rev	CTCATGCAGACCACACAAGG<br>TAATACGACTCACTATAGGGAAAATCACATGGGGATAGCA	GGGAAAATCACATGGGGATAGCACTACTAAAATTAATTTTACACATTAGGGCTCTTCCATATAGGCAGCTCTCCCTAGCATTGTTCACTGTACACTCGATCGTACTCCGCGTGGCCTCGGTGAAAATGTGGTGGCTCTTTCAAGTCCTCCCTAATGTTACACACTGATTAAAGATTGCTATGTGAGATTAAAGTT<br>AACTACATCTACTTGTGCTATGTAGTTACGAGAATTCATTCTGCACAAGAGTAGACTATATATCGTAAACGGAAAAGCGAAAACGTTTATATAGCCCATCTGCCTTGTGTGGTCTGCATGAG
5´-leader reporter	_fwd<br>_rev	ATTAAAGGTTTATACCTTCCCAG<br>TAATACGACTCACTATAGGGAGTGCTTTTGGCGAAGAAGG	GGGAGTGCTTTTGGCGAAGAAGGAGAATAGGGTTGGCACCAGCAGCGCACTTTGAATCTTGTAATCCTGAAGGCTCCTCAGAAACAGCTCTTCTTCAAATCTATACATTAAGACGACTCGAAATCCACATATCAAATATCCGAGTGTAGTAAACATTCCAAAACCGTGATGGAATGGAACAACACTTAAAATCGC<br>AGTATCCGGAATGATTTGATTGCCAAAAATAGGATCTCTGGCATGCGAGAATCTCACGCAGGCAGTTCTATGAGGCAGAGCGACACCTTTAGGCAGACCAGTAGATCCAGAGGAGTTCATGATCAGTGCAATTGTCTTGTCCCTATCGAAGGACTCTGGCACAAAATCGTATTCATTAAAACCGGGAGGTAGATG<br>AGATGTGACGAACGTGTACATCGACTGAAATCCCTGGTAATCCGTTTTAGAATCCATGATAATAATTTTTGGATGATTGGGAGCTTTTTTGCACGTTCAAAATTTTTTGCAACCCCTTTTTGGAAACGAACACCACGGTAGGCTGCGAAATGCCCATACTGTTGAGCAATTCACGTTCATTATAAATGTCGTT<br>CGCGGGCGCAACTGCAACTCCGATAAATAACGCGCCCAACACCGGCATAAAGAATTGAAGAGAGTTTTCACTGCATACGACGATTCTGTGATTTGTATTCAGCCCATATCGTTTCATAGCTTCTGCCAACCGAACGGACATTTCGAAGTACTCAGCGTAAGTGATGTCCACCTCGATATGTGCATCTGTAAAAGC<br>AATTGTTCCAGGAACCAGGGCGTATCTCTTCATAGCCTTATGCAGTTGCTCTCCAGCGGTTCCATCTTCCAGCGGATAGAATGGCGCCGGGCCTTTCTTTATGTTTTTGGCGTCGGATCCCAATAAAACAAGAAAAACAAACATTGTTCGTTTAGAGAACAGATCTACAAGAGATCGAAAGTTGGTTGGTTTGTT<br>ACCTGGGAAGGTATAAACCTTTAAT
			**Legend**<br>GGG**NNNNNNNNNNNNNNNNNNNNNNNNNNNNNNNNNNNNNNNNNNNNNN**TTTTTTTTTTTTTTTTTTTT<br>GGG = transcription start, **NN** = target region , TTT = Oligo(T) for DIG-labeling

Northern Blot standard

Name		Oligonucleotides 5´-3´	Standard sequence 5´-3´
1-40 (Tornado)	_fwd<br>_rev	TAATACGACTCACTATAGGGAAGGTATAAACCTTTAATAAAAACCGCGGTCGGCGTGGACTGTAGAACCATGCCGAGTGCGGCCGCAAGTTGGTTGGTTAGTTACCTGGG<br>CCCAGGTAACTAACCAACCAACTTGCGGCCGCACTCGGCATGGTTCTACAGTCCACGCCGACCGCGGTTTTTATTAAAGGTTTATACCTTCCCTATAGTGAGTCGTATTA	GGGAAGGTATAAACCTTTAATAAAAACCGCGGTCGGCGTGGACTGTAGAACCATGCCGAGTGCGGCCGCAAGTTGGTTGGTTAGTTACCTGGG
			**Legend**<br>GGG**NNNNNNNNNNNNNNNNNN**AAAAACCGCGGTCGGCGTGGACTGTA**GA**ACCATGCCGAGTGCGGCCGCAA**NNNNNNNNNNNNNNNNNNNNNNNNNNNN**<br>GGG = transcription start, **NN** = antisense region, flexible linker, Tornado stem loop , **GA** = circ-junction, **N** = T->A (for extended T-stretches)

RNase H cleavage

Name	Oligonucleotide 5´-3´
1-75	GTTCTCTAAACGAACTTGCGG
5´-leader reporter	GCCCATATCGTTTCATAGCT

Antisense oligonucleotides (ASOs)

Name	Oligonucleotide 5´-3´ (2´-OMe or 2´-MOE modified)
CTR1	CGCACCACGCCACGCGTAGA
CTR2	ACGGGAGGATACCCGCTGTC
1-45	CGAAAGTTGGTTaaTTTAAT
56-75	GTTCGTTTAGAGAACAGATC

RT-qPCR Primer

Name	Oligonucleotide 5´-3´
Firefly fwd	GAGGTTCCATCTGCCAGGTA
Firefly rev	CCGGTATCCAGATCCACAAC
Renilla fwd	AACGCGGCCTCTTCTTATTT
Renilla rev	ATTTGCCTGATTTGCCCATA

Legend for Supplementary Table S1

Supplementary Table S1.
List of oligonucleotides and RNA sequences used in this study
Oligonucleotides used for circRNA production (Tornado-based and *in vitro* production), for Northern Blot analysis, RNase H cleavage, comparison to ASOs (2´-OMe or 2´-MOE modified), and as primers for RT-qPCR.
